# A systematic analysis of natural *α*-glucosidase inhibitors from flavonoids of *Radix scutellariae* using ultrafiltration UPLC-TripleTOF-MS/MS and network pharmacology

**DOI:** 10.1186/s12906-020-2871-3

**Published:** 2020-03-06

**Authors:** Le Wang, Nana Tan, Huan Wang, Jingbo Hu, Wenbo Diwu, Xiaoling Wang

**Affiliations:** 1 Shaanxi Key Laboratory of Phytochemistry, College of Chemistry and Chemical Engineering, Baoji University of Arts and Sciences, High-tech Avenue 1#, Baoji, 721013 China; 2 College of Computer Science and Technology, Baoji University of Arts and Sciences, Baoji, 721013 China; 3 College of Electronic and Electrical Engineering, Baoji University of Arts and Sciences, Baoji, 721013 China; 4 Department of Physics, Center for Nonlinear Complex Systems, School of Physics and Astronomy, Yunnan University, Kunming, 650091 China

**Keywords:** *Radix scutellariae*, Flavonoids, *α*-Glucosidase inhibitors, Ultrafiltration, LC-MS, Network pharmacology

## Abstract

**Background:**

Flavonoids from plant medicines are supposed to be viable alternatives for the treatment of type 2 diabetes (T2D) as less toxicity and side effects. *Radix scutellariae* (*RS*) is a widely used traditional medicine in Asia. It has shown great potential in the research of T2D. However, the pharmacological actions remain obscured due to the complex chemical nature of plant medicines.

**Methods:**

In the present study, a systematic method combining ultrafiltration UPLC-TripleTOF-MS/MS and network pharmacology was developed to screen *α*-glucosidase inhibitors from flavonoids of *RS*, and explore the underlying mechanism for the treatment of T2D.

**Results:**

The *n*-butanol part of ethanol extract from *RS* showed a strong *α*-glucosidase inhibition activity (90.55%, IC_50_ 0.551 mg/mL) against positive control acarbose (90.59%, IC_50_ 1.079 mg/mL). A total of 32 kinds of flavonoids were identified from the extract, and their ESI-MS/MS behaviors were elucidated. Thirteen compounds were screened as *α*-glucosidase inhibitors, including viscidulin III, 2′,3,5,6′,7-pentahydroxyflavanone, and so on. A compound-target-pathway (CTP) network was constructed by integrating these *α*-glucosidase inhibitors, target proteins, and related pathways. This network exhibited an uneven distribution and approximate scale-free property. Chrysin (*k* = 87), 5,8,2′-trihydroxy-7-methoxyflavone (*k* = 21) and wogonin (*k* = 20) were selected as the main active constituents with much higher degree values. A protein-protein interaction (PPI) weighted network was built for target proteins of these *α*-glucosidase inhibitors and drug targets of T2D. PPARG (*C*_*d*_ = 0.165, *C*_*b*_ = 0.232, *C*_*c*_ = 0.401), ACACB (*C*_*d*_ = 0.155, *C*_*b*_ = 0.184, *C*_*c*_ = 0.318), NFKB1 (*C*_*d*_ = 0.233, *C*_*b*_ = 0.161, *C*_*c*_ = 0.431), and PGH2 (*C*_*d*_ = 0.194, *C*_*b*_ = 0.157, *C*_*c*_ = 0.427) exhibited as key targets with the highest scores of centrality indices. Furthermore, a core subnetwork was extracted from the CTP and PPI weighted network. Type II diabetes mellitus (hsa04930) and PPAR signaling pathway (hsa03320) were confirmed as the critical pathways.

**Conclusions:**

These results improved current understanding of natural flavonoids on the treatment of T2D. The combination of ultrafiltration UPLC-TripleTOF-MS/MS and network pharmacology provides a novel strategy for the research of plant medicines and complex diseases.

## Background

Type 2 diabetes (T2D) is one of the most serious chronic metabolic disorders characterized by persistent hyperglycemia. It accounts for more than 90% of all diabetes [1], and is directly linked to pathogenic consequences in the eyes, kidney, and cardiovascular diseases [2]. Natural products with less side effects have been used to treat diabetes for thousands of years [3]. Previously, various natural products were found to exhibit anti-diabetic effects, such as herbal formulas, fruits, vegetables, spices, or natural beverages [4]. These remedies are more accessible and affordable than modern pharmaceuticals [5].

*Radix scutellariae* (*RS*) is the dried root of *Scutellaria baicalensis* [6]. It is widely used as herbal medicine in Asia for thousands of years [7]. This medicine has various therapeutic functions, including antitumor, cardiovascular, neuroprotective and anti-inflammatory effects [6, 8, 9]. A growing body of exciting evidences also indicated an antidiabetic effect of *RS*. For instance, water extract of *RS* showed a reduction of body weight and blood triglyceride in type 2 diabetic db/db mice [10]. Methanol extract of *RS* had strong *α*-glucosidase inhibition [11]. Another traditional medicine coptis together with *RS* demonstrated potent anti-hyperglycemic effect on diabetic rats [12]. In addition, ethanolic extract of *RS* was found to enhance the antidiabetic effect of metformin, and increase pancreatic insulin content as well as improving the lipid profile in diabetic Wistar rats [13]. These reports suggested great potential of *RS* in the drug discovery of T2D.

The *α*-glucosidase is an exo-type carbohydrate enzyme that catalyzes the liberation of *α*-glucose from the non-reducing end of the carbohydrates. It locates in the brush border surface membrane of the small intestinal cells. This enzyme accelerates glucose reabsorption in the intestine [14]. Inhibition of *α*-glucosidase could delay the digestion, absorption of carbohydrates, and suppress postprandial hyperglycemia [15]. Natural *α*-glucosidase inhibitors have presented viable alternatives to the treatment of T2D as fewer toxicity and adverse effects [16]. More than one hundred herbal medicines have exhibited great potency in *α*-glucosidase inhibition and equivalent efficacies to synthetic drugs in managing diabetes [17].

Numerous compounds of natural origin are considered as models for drug discovery of T2D, such as flavonoids, polyphenols, terpenoids, alkaloids, saponins, quinones [18]. Flavonoids are a group of natural polyphenolic derivatives that widely exist in traditional medicines [19]. In recent years, considerable portions of natural flavonoids displayed anti-diabetic effects, including quercetin, rutin, naringin, baicalein [20–22]. Many flavonoids were also found in *RS*, such as baicalein, baicalin, wogonin, wogonoside, and so on [23]. A part of these compounds were reported to exhibit *α*-glucosidase inhibition [22]. Therefore, *RS* is considered as a source of natural *α*-glucosidase inhibitors.

The identification of the pharmacological profile of natural products is always a challenging task. Ultrafiltration method has attracted much attention in the screening and analysis of bioactive compounds from botanical extracts [24]. It has the advantage of high-speed and high-reliability, which facilitates the separation of ligand-receptor complexes for unbound compounds [25, 26]. For instance, Zhang et al. established an ultrafiltration LC-MS method for screening and characterizing thrombin inhibitors from Rhizoma. Wang et al. applied ultrafiltration LC-MS combined with reverse phase-medium pressure liquid chromatography for screening and isolation potential *α*-glucosidase inhibitors from *RS* [27]. However, these studies mainly focused on the screening of bioactive molecules. Further researches are urgently needed to elucidate the underlying mechanism.

Molecular mechanism of natural products is always difficult and confused as the complex chemical nature [28, 29]. Recently, network pharmacology approach, also known as system pharmacology, has emerged as a powerful tool to solve the problem [30, 31]. This methodology holds a significant potential for extracting biological information from large amounts of chemical data [32], and enables to predict the target profiles and pharmacological actions of herbal compounds [33, 34]. Chen et al. constructed a multi-parameter network model on the basis of three important parameters to tentatively explain the anti-fibrosis mechanism of herbal medicine *Sophora flavescens* [35]. Luo et al. used systems pharmacology strategies for anti-cancer drug discovery based on natural products [36]. Gogoi et al developed a network pharmacology-based virtual screening of natural products from *Clerodendrum* species for identification of novel anti-cancer therapeutics [37]. These studies demonstrated that network pharmacology approach had real potential in the mechanism research of natural products [38, 39].

In the present study, we developed a systematic method to screen *α*-glucosidase inhibitors from plant flavonoids and explore the underlying mechanism. Ultrafiltration UPLC-TripleTOF-MS/MS was used to identify flavonoids from the *RS* extract and screen potential *α*-glucosidase inhibitors. Network pharmacology was applied to investigate the interrelationships between these compounds, related target proteins and pathways. Several networks were constructed, and a series of topological characteristics were calculated to determine the main active constituents, key targets and critical pathways.

## Methods

### Materials and reagents

Crude *Radix scutellariae* was purchased from Baoji Medicinal Material Company (Shaanxi, China). The plant species was authenticated by Prof. Xiaomei Wang from Shaanxi Key Laboratory of Phytochemistry in Baoji University of Art and Sciences. Wogonin (HW158604), baicalin (HB158602), wogonoside (HW158601), oroxylin A (HB158728), chrysin (C110078), skullcapflavone II (HA062620), oroxylin A^− 7^-O-*β*-D-glucuronopyranoside (HO158605), baicalein-6-O-*β*-D-glucuronopyranoside (XB161661), and chrysin-7-O-*β*-D-glucuronopyranoside (HA061609) were obtained from Chenguang Biotech Co. Ltd. (Shaanxi, China) with a purity higher than 98%. The *α*-Glucosidase (from *Saccharomyces cerevisiae*) was purchased from Sigma-Aldrich (St. Louis, MO, USA). Acarbose and *p*-nitrophenyl-*α*-D-glucopyranoside (*α*-pNPG) were acquired from Aladdin Industrial Corporation (Shanghai, China). Methanol of HPLC grade was supplied by Merck (Darmstadt, Germany). Formic acid (HPLC grade) was purchased from Tedia (Fairfield, OH, USA). Ultrapure water was obtained using a Milli-Q purification system (Millipore Co., USA). All other reagents were analytically pure.

### Standards and sample preparation

Reference substances were accurately weighed and dissolved in methanol (5 μg/mL). The solutions were stored at 4 °C until use. Dried *Radix scutellariae* powders (100 g) were passed through 100-mesh sieves, then orderly extracted with *n*-hexane, chloroform, 70% ethanol by heating reflux for 2–3 h, three times. Then, the 70% ethanol solution was leached with *n*-butanol (saturated by water). The solvents were removed by evaporation in vacuo, and the extracts were stored at − 20 °C until required, thawed at room temperature, dissolved in methanol (1 mg/mL). Finally, the solution was filtered with 0.22 μm Millipore filter membrane, and used directly for LC-MS.

### *α*-Glucosidase inhibition assay

The *α*-glucosidase inhibitory activity was evaluated based on the slightly modified method of the literature [40]. The assay mixture (160 μL) contained 20 μL of phosphate buffer (0.1 M, pH 7.0) in 96-well plates, 20 μL of enzyme solution (0.1 U /mL *α*-glucosidase in phosphate buffer), 20 μL of sample in phosphate buffer with different concentrations to be mixed and incubated at 37 °C for 15 min. Then, the reaction was initiated by adding 20 μL of *α*-*p*NPG (2.5 mM in phosphate buffer). After 15 min at 37 °C, the reaction was stopped by adding 80 μL Na_2_CO_3_ (0.2 M) solution. Amount of released PNP (4-nitrophenol) was quantified by a microplate reader at the absorbance of 405 nm. The inhibitory rates (%) were calculated as follows:

$$ \mathrm{Inhibition}\%=1-\left[\left({\mathrm{A}}_{\mathrm{s}}-{\mathrm{A}}_{\mathrm{c}}\right)/{\mathrm{A}}_{\mathrm{b}}\right]\times 100 $$

Where the symbol ‘A_s_’ is the absorbance of the test sample; ‘A_c_’ is the absorbance of the sample contrast (without enzyme solution); and ‘A_b_’ is the absorbance of the blank (without tested sample). All reactions were conducted in three replications and acarbose was used as positive control. The half maximal inhibitory concentration of the test sample (IC_50_) was calculated using the modified Karber’s method.

### Screening of *α*-glucosidase inhibitors from *RS*

A 2 μL aliquot of *n*-butanol part of ethanol extract from *RS* (50 mg/mL) was incubated with 8 μL of *α*-glucosidase (100 μM, dissolved in 10 mM ammonium acetate buffer, pH 6.86) for 30 min at 37 °C. After incubation, each sample was filtered through a Vivaspin 2 concentrator (MWCO 10 kDa, Sartorius, Göttingen, Germany) at 10,000 g for 10 min. Then, the filter was washed three times with 200 μL ammonium acetate buffer (PH 6.86) to remove the unbound compounds. The bound ligands were released by adding 200 μL of methanol/water mixtures (1:1, *v/v*) adjusted with acetic acid to pH 3.30, followed by centrifugation at 10,000 g for 15 min. This procedure was repeated three times. A control experiment in which *α*-glucosidase omitted was also carried out before each screening experiment. The released ligands were then analyzed by LC-MS.

### UPLC-TripleTOF-MS/MS analysis

Chromatographic separations were achieved on LC-20AD^XR^ (Shimadzu, Tokyo, Japan) coupled with a Shim-pack XR-ODS column (100 mm × 2.0 mm, 2.2 μm, Shimadzu). The mobile phases consisted of eluent A (0.1% formic acid in water, *v/v*) and eluent B (0.1% formic acid in methanol, *v/v*). The gradient elution program was set as follows: 10 to 48% B from 0 to 8 min, holding for 6 min, 48 to 100% B from 14 to 20 min. After holding 100% B for next 5 min, the ratio was returned to its starting condition. The injection volume was 20 μL (20 μg/mL) at a flow rate of 0.3 mL/min. The column was maintained at 40 °C. MS analysis was performed on a TripleTOF 4600 mass analyzer (AB SCIEX, USA) equipped with electrospray ionization (ESI) source. The instrument was operated in positive ESI mode with a declustering potential voltage (DP) of 100 V and ionspray voltage of 5.5 KV. The nebulization temperature was 550 °C. GS1, GS2 and curtain gas were maintained at 55, 55 and 30 psi, respectively. Collision energy was 10 eV for MS and 50 eV for MS/MS. An automated calibration delivery system (CDS) was applied to regulate the MS and the MS/MS. The constituents were identified by the comparison with reference standards, the accurate molecular weights (with a mass tolerance of ±5 ppm), as well as the MS/MS fragment patterns. The operations, acquisition, and analysis of data were monitored by Analyst TF 1.7 (AB SCIEX, Concord, Canada) and PeakView 2.0 (AB SCIEX, Concord, Canada).

### Collection of target proteins and pathways enrichment analysis

Target proteins of the *α*-glucosidase inhibitors from *RS* were collected using SuperPred (http://prediction.charite.de/) and DrugBank (https://www.drugbank.ca/). The target prediction is based on the similarity distribution among the targets’ ligands. The distributions are utilized for estimating individual thresholds and probabilities for a specific target. By means of these individual thresholds and probabilities, the input compound was screened against a database containing about 341,000 compounds, 1800 targets and 665,000 compound-target interactions [41]. Information of all the targets was uniformed by Uniprot (http://www.uniprot.org/). Pathway analysis was applied to these proteins by DAVID 6.8 (https://david.ncifcrf.gov/). The queried species was *Homo sapiens*. Raw *P*-values were adjusted using Benjamini & Hochberg procedure [42]. Pathways with adjusted *P*-values less than 0.05 were considered as significant.

### Construction of networks for the *α*-glucosidase inhibitors from *RS*

A complex network analysis was performed on the collected data for further interpretation. First, a compound-target-pathway (CTP) network was constructed to screen main active components from *RS*. This network consisted of numerous nodes and edges. Nodes represented the *α*-glucosidase inhibitors from *RS*, corresponding target proteins and related pathways, respectively. Edges referred to interactions between them. If a protein was the hit target of particular inhibitor, or involved in any pathways, connections were made between these elements.

Subsequently, we collected therapeutic targets of T2D from TTD database (https://db.idrblab.org/ttd/) [43], and integrated with targets of the *α*-glucosidase inhibitors into a protein-protein interaction (PPI) weighted network*.* This network was designed to evaluate the closeness of interaction between *RS* and T2D. Interactions between the two groups of proteins were calculated by Search Tool for the Retrieval of Interacting Genes/Proteins (STRING, https://string-db.org/) [44]*.* STRING uses a scoring mechanism to give a comprehensive score to the results obtained by these different methods, including experimental data, data mined from PubMed abstract text, database data, and results predicted by bioinformatics methods. A weighted protein-protein interaction (PPI) network was constructed on the basis of these data. Nodes indicated the proteins, and that connections represented interactions between them with scores higher than 0.7.

Moreover, key nodes of the PPI network and their neighbor nodes were extracted, as well as the directly connected *α*-glucosidase inhibitors and related pathways. These elements were reconstructed as a core subnetwork to explore the underlying pharmaceutical mechanism of *RS*. Construction and visualization of all the networks were performed by Pajek ver. 2.00 (Batagelj and Mrvar, 2009).

### Statistical and topological analysis of the network

To interpret the behavior of the *α*-glucosidase inhibitors from *RS* and T2D, several topological parameters of the network were analyzed (Table 1). The degree *k*_*i*_ is the number of its connections attached to a given node *i*. The directly linked nodes are called neighbors of node *i*. Mean value of *k* of all nodes is defined as average degree ⟨*k*⟩. Degree distribution is the proportion of randomly selected nodes with specific number of connections, and denoted as *P(k)*. Average path length (*L*) refers to the mean distance between each pairs of nodes, which measures the overall navigability of a network. The diameter (*D)* is the maximum distance between any pair of nodes.

**Table 1:** Definitions of the topological parameters used in the network analysis

Statistical characteristic	Symbol	Equation ^**a**^
Degree	*k*	${k}_i=\sum \limits_{j=1}^N{e}_{ij}$
Average degree	*<k>*	$<k>=\frac{1}{N}{\sum}_{i=1}^N{k}_i$
Average path length	*L*	$L=\frac{1}{N\left(N-1\right)}\sum \limits_{i\ne j}{d}_{ij}$
Diameter	*D*	*D* = max {*d*_*ij*_}
Node strength	*s*	${s}_i=\sum \limits_{j\in {N}_i}{w}_{ij}$
Dispersion of weight distribution	*Y*	${Y}_i=\sum \limits_{j\in {N}_i}{\left[\frac{w_{ij}}{s_i}\right]}^2$
Degree centrality	*C*_*d*_	${C}_d=\frac{k_i}{N-1}$
Betweenness centrality	*C*_*b*_	${C}_b=\sum \limits_{j\left(<k\right)}^N\sum \limits_k^N\frac{g_{jk}(i)}{g_{jk}}$
Closeness centrality	*C*_*c*_	${C}_c=\frac{N-1}{\sum \limits_{j=1}^N{d}_{ij}}$

^a^*N* is the total number of all nodes in the network; *e*_*ij*_ is the numbers of edges from node *i* to *j*; *d*_*ij*_ is the shortest path length from node *i* to *j*; *g*_*jk*_ is the numbers of geodesics connecting nodes *j* and *k*; *N*_*i*_ is the neighbor collection of node *i*; *W*_*ij*_ is the edge weight between node *i* and *j*

Centrality measures the relative influence of a node within the overall architecture of a network. In this study, three centrality metrics were comprehensively evaluated. Each of them focused on specific influence of a node on other nodes. Degree centrality (*C*_*d*_) indicates the proportion of other nodes adjacent to a node, representing the immediate influence that the closest nodes produce on the corresponding vertex. Betweenness centrality (*C*_*b*_) refers to the total number of shortest paths going through a node, which directly reflects the influence of a node has on the spread of information through the network. Closeness centrality (*C*_*c*_) is the number of other nodes divided by the sum of distances between one node and the others, reflecting how close a node is to others. The statistical analysis was performed with MATLAB 2016a (The MathWorks Inc., Natick, MA, USA).

## Results

### Identification of flavonoids from *Radix Scutellariae*

The *n*-butanol part of ethanol extract from *Radix Scutellariae* were analyzed by UPLC-TripleTOF-MS/MS. A total of 32 kinds of flavonoids were identified within 5 ppm mass tolerance. Nine of them (compound 10, 11, 12, 14, 16, 24, 26, 28, 29) were confirmed by the reference standards, and the others by fragmentation analysis. The identification results were also compared with those from previous studies to ensure the accuracy. Detailed MS data of these compounds are listed in Table 2, and the MS/MS fragmentation patterns of typical flavonoids from *RS* are shown in Additional file 1.

**Table 2:** Characterization of flavonoids from *Radix Scutellariae* extracts by UPLC-TripleTOF-MS/MS

No.	Compound ^a^	*t*_R_ (min)	Formula	Measured m/z ^b^	Error ^c^ (ppm)	MS^2^ (*m/z*)
1	2′,3,5,6′,7-Pentahydroxyflavanone	5.80	C_15_H_12_O_7_	305.0659	1.00	153.0079, 123.0366, 97.0212
2	5,2′6’-Trihydroxy-7,8-dimethoxyflavone-2′-O-*β*-D-glucopyranoside	8.69	C_23_H_24_O_12_	493.1336	−0.90	331.0841, 316.0528, 298.0487, 287.0291
3	2′,5,6′,7-Tetrahydroxyflavane	9.33	C_15_H_12_O_6_	289.0709	0.80	169.0200, 153.0076, 147.0345, 134.9982
4	Chrysin-7-O-*β*-D-glucopyranoside	9.55	C_21_H_20_O_9_	417.1179	−0.30	307.0808, 297.0547, 279.0451, 267.0465
5	Viscidulin III	9.58	C_17_H_14_O_8_	347.0764	0.70	314.0373, 289.0361, 286.0436, 233.0423
6	2′,6′,7-Trihydroxy-5-methoxyflavanone	9.69	C_16_H_14_O_6_	303.0859	−1.40	167.0340, 152.0095, 123.0445, 107.0489
7	Baicalein-7-O-*β*-D-glucopyranoside	10.79	C_21_H_20_O_10_	433.1133	0.90	271.0555, 253.0444, 197.0546, 169.0102
8	Dihydroxybaicalein	11.00	C_15_H_12_O_5_	273.0760	0.90	169.0112, 123.0070, 103.0529
9	5,7,2′-Trihydroxy-6′-methoxyflavone	11.33	C_16_H_12_O_6_	301.0700	−2.20	250.9845, 241.0439, 153.0148, 139.0000
10	Baicalin *	11.33	C_21_H_18_O_11_	447.0925	0.70	271.0583, 253.0482, 225.0534, 1,690,121
11	Baicalein-6-O-*β*-D-glucuronopyranoside *	12.57	C_21_H_18_O_11_	447.0926	0.90	327.0583, 271.0590, 253.0515, 184.0556
12	Chrysin-7-O-*β*-D-glucuronopyranoside *	13.07	C_21_H_18_O_10_	431.0971	−0.40	255.0683, 187.0802, 1,530,556, 103.0534
13	5,6,7-Trihydroxy-8-methoxyflavone-7-O-*β*-D-glucuronopyranoside	13.14	C_22_H_20_O_12_	477.1027	−0.10	301.0752, 286.0516, 199.0233, 184.0029
14	Oroxylin A-7-O-*β*-D-glucuronopyranoside *	13.73	C_22_H_20_O_11_	461.1071	−1.60	285.0748, 270.0550, 242.0571, 168.0048
15	5,7,2′-Trihydroxy-6-methoxyflavone-7-O-*β*-D-glucuronopyranoside	13.87	C_22_H_20_O_12_	477.1029	0.30	301.0735, 286.0499, 183.9976, 157.0433
16	Wogonoside *	14.14	C_22_H_20_O_11_	461.1069	−2.00	285.0748, 270.0550, 242.0571, 149.1111
17	4′-Hydroxywogonin	16.22	C_16_H_12_O_6_	301.0703	−1.20	286.0501, 184.0006, 156.0054, 137.9944
18	6-Methoxynaringenin	16.85	C_16_H_14_O_6_	303.0865	0.60	168.0022, 147.0416, 135.0801, 129.0299
19	5,6,7-Trihydroxy-4′-methoxyflavanone	16.86	C_16_H_14_O_6_	303.0861	−0.70	147.0423, 135.0801, 129.0299, 107.0491
20	2′,5,6′,7-Tetrahydroxy-8-methoxyflavone	16.86	C_16_H_12_O_7_	317.0658	0.70	209.0372, 147.0453, 129.0344, 123.0368
21	5,8,2′-Trihydroxy-6,7-dimethoxyflavone	16.86	C_17_H_14_O_7_	331.0811	−0.40	301.0164, 239.0294, 183.0857, 147.0425
22	Oroxylin A-7-O-*β*-D-glucuronide methyl ester	17.42	C_23_H_22_O_11_	475.1231	−0.80	285.0784, 271.0634, 253.0525, 225.0554
23	5,8,2′-Trihydroxy-7-methoxyflavone	17.74	C_16_H_12_O_6_	301.0709	0.80	286.0456, 168.0050, 140.0096, 121.0289
24	Skullcapflavone II *	17.91	C_19_H_18_O_8_	375.1077	0.70	345.0560, 327.0460, 227.0541, 212.0289
25	Dihydrooroxylin A	18.22	C_16_H_14_O_5_	287.0916	0.70	272.0570, 183.0258, 168.0043, 140.0092
26	Wogonin *	18.32	C_16_H_12_O_5_	285.0764	2.30	270.2520, 252.0420, 241, 0409, 179.0462
27	5,8-dihydroxy-6,7-dimethoxyflavone	18.43	C_17_H_14_O_6_	315.0867	1.20	285.0382, 257.0436, 197.0589, 182.9920
28	Chrysin *	18.62	C_15_H_10_O_4_	255.0654	0.80	153.0192, 129.0342, 103.0547
29	Oroxylin A *	18.71	C_16_H_12_O_5_	285.0760	0.90	270.0511, 168.0051, 140.0097, 112.0151
30	Tenaxin I	19.18	C_18_H_16_O_7_	345.0971	0.60	315.0528, 297.0426, 272.0299, 197.0095
31	Moslosooflavone	19.43	C_17_H_14_O_5_	299.0911	−1.00	284.0606, 283.0583, 255.0621, 238.0570
32	Oroxylin A-7-O-*β*-D-glucuronopyranoside butyl ester	19.52	C_26_H_28_O_11_	517.1694	−2.00	285.0532, 270.0317

^a^ Compounds were identified by the comparison with exact mass (< 5 ppm), reference standards (indicated by an asterisk), as well as the MS/MS fragmentation patterns (Ref. [7, 8, 45–54]); ^b^ Measured m/z of peak [M + H]^+^; ^c^ Mass accuracy between the calculated m/z and measured m/z of peak [M + H]^+^

The identified flavonoids contained 20 aglycones (compound 1, 3, 5, 6, 8, 9, 17–21, 23–31) and 12 glycosides (compound 2, 4, 7, 10–16, 22, 32). Compound 1, 3, 8, 28 belonged to aglycones, substituted by several hydroxyl groups. Compound 28 showed [M + H]^+^ peak at *m/z* 255.0654 (Additional file 1a). This protonated molecular ion yielded the product ions at *m/z* 153.0192, 103.0547 in the MS/MS spectra. The two ions were attributed to the ^1,3^A^+^ and ^1,3^B^+^, indicating the occurrence of two OH groups in A-ring and none OH in B-ring. It was consistent with the report of chrysin by Luo et al [7]. Hence, compound 28 was tentatively identified as chrysin, which was further confirmed by the standard. Similarly, compound 1, 3, 8 were identified as 2′,3,5,6′,7-pentahydroxyflavanone, 2′,5,6′,7-tetrahydroxyflavane, and dihydrobaicalein, respectively [45, 46].

Compound 5, 6, 9, 17–21, 23–27, 29–31 belonged to the methoxylated flavonoid aglycones. They exhibited a characteristic ion (15*n Da) due to the loss of CH_3_ radicals. Protonated molecular ion of compound 26 was observed at *m/z* 285.0764 (Additional file 1b). The sole flavone aglycone easily gave a prominent ion [M + H-15]^+^ at *m/z* 270.2520, originated from losing one CH_3_ (15 Da). It also lost a CHO (29 Da) from C-ring, and produced the fragment at *m/z* 241.0494. In addition, the neutral loss of H_2_O (18 Da) from *m/z* 270.2520 produced the ions at *m/z* 252.0420 and 179.0462. Diagnostic fragment ions originated from Retro-Diels-Alder (RDA) reaction are often helpful to the structural determination of A- and B-ring substitution patterns [45]. Our data showed fragment ions ^1,3^A^+^ (*m/z* 168.0076) and ^1,3^B^+^ (*m/z* 103.0523), originated from the cleavage of the bond at position 1/3 of C-ring. It was also annotated by Ma et al. [47], and that compound 26 was finally identified as wogonin.

Compound 17 showed [M + H]^+^ peak at *m/z* 301.0703 (Additional file 1c). MS/MS spectra of this compound exhibited a methoxylated flavone characteristic loss of CH_3_ (15 Da), resulting in a product ion at *m/z* 286.0501. Besides, the parent ion *m/z* 301.0703 also yielded the ions at *m/z* 184.0006, 156.0054, 137.9944, and 119.0452. The product ion *m/z* 184.0006 was attributed to the ^1,3^A^+^, indicating that the substituent groups of two OH and an OCH_3_ were located in A-ring. The ion *m/z* 156.0054 was produced by the neutral loss of CO and H_2_O from the ^1,3^A^+^. This compound was finally identified as 4′-hydroxywogonin [46]. Compound 23 (Additional file 1d) exhibited a same characteristic loss of CH_3_ (15 Da) at *m/z* 286.0456. Moreover, other RDA fragments from the fragment ion, ^1,3^A^+^ at *m/z* 168.0050 and ^1,4^A^+^ at *m/z* 140.0096, could also be observed. It was identified as 5,8,2′-trihydroxy-7-methoxyflavone, the isomer of compound 17. Identification of other compounds in this group was also conducted by comparison with previous reports, including compound 5, 6, 9 [7, 45, 48], compound 18–21 [49, 50], compound 25 [51], compound 27 [52], compound 30–31 [8].

Compound 2, 4, 7, 10–16, 22, 32 belonged to flavonoid glycosides, glycosylated in different positions. Neutral loss of glucuronic acid (176 Da) or glucose (162 Da) is common in flavone glycoside, as the O-glucosylic bond is easily cleaved to generate aglycone. Reference standards of compound 10, 11, 12, 14, and 16 showed a fragment ion at [M + H-176]^+^ due to the loss of glucuronic acid. These compounds could be further distinguished by the fragment of residual aglycone. Compound 14 (Additional file 1e) and compound 16 (Additional file 1f) were a pair of isomers. They both went through the loss of glucuronic acid (176 Da), and produced the aglycone ions wogonin (8-OCH_3_) and oroxylin A (6-OCH_3_), respectively. Furthermore, they both exhibited a base peak [M + H-176-CH_3_] at *m/z* 270.0494. However, these two compounds could be distinguished by the relative abundances of the losses of CO and CHO from the parent ion [M + H-176-CH_3_]. In the MS/MS of compound 16, the relative abundance of ion [M + H-176-CH_3_-CO] at *m/z* 242.0598 was lower than [M + H-176-CH_3_-CHO] at *m/z* 241.0494, since the loss of CHO could produce a more stable *p*-quinoid skeleton. Nevertheless, it was contrary to compound 14. Finally, compound 14 was identified as oroxylin A-7-O-*β*-D-glucuronopyranoside, and compound 16 was wogonoside.

Compound 12 also exhibited the characteristic fragment ion [M + H-176] at *m/z* 255.0683. Moreover, other RDA fragments from the aglycone ion of chrysin (^1,3^A_0_^+^ at *m/z* 153.0186 and ^1,3^B_0_^+^ at *m/z* 103.0558) were observed. This compound was identified as chrysin-7-O-*β*-D-glucuronopyranoside, which was supported by the report by Luo et al [7]. Identification of other compounds in this group was according to previous studies, including compound 2 and 4 [7], compound 7 [53], compound 13 and 15 [8], compound 22 and 32 [54]. However, these results are only based on LC-MS/MS, which might be limited by various factors. More reference standards and analytical tools would be used to check the accuracy of identification in our next study.

### Potential *α*-glucosidase inhibitors, target proteins and related pathways

An in vitro *α*-glucosidase inhibition assay was performed on the *n*-butanol part of ethanol extract from *RS* (Additional file 2). It showed higher *α*-glucosidase inhibitory activity (IC_50_ = 0.551 mg/mL) than the positive control (IC_50_ = 1.079 mg/mL). The inhibition rate reached 90.55% at the concentration of 2.34 mg/mL, whereas that of the positive control was 90.59% at 15 mg/mL. These data indicated that the crude extract of *RS* was much more potent than acarbose on *α*-glucosidase inhibition.

**Fig. 1:**
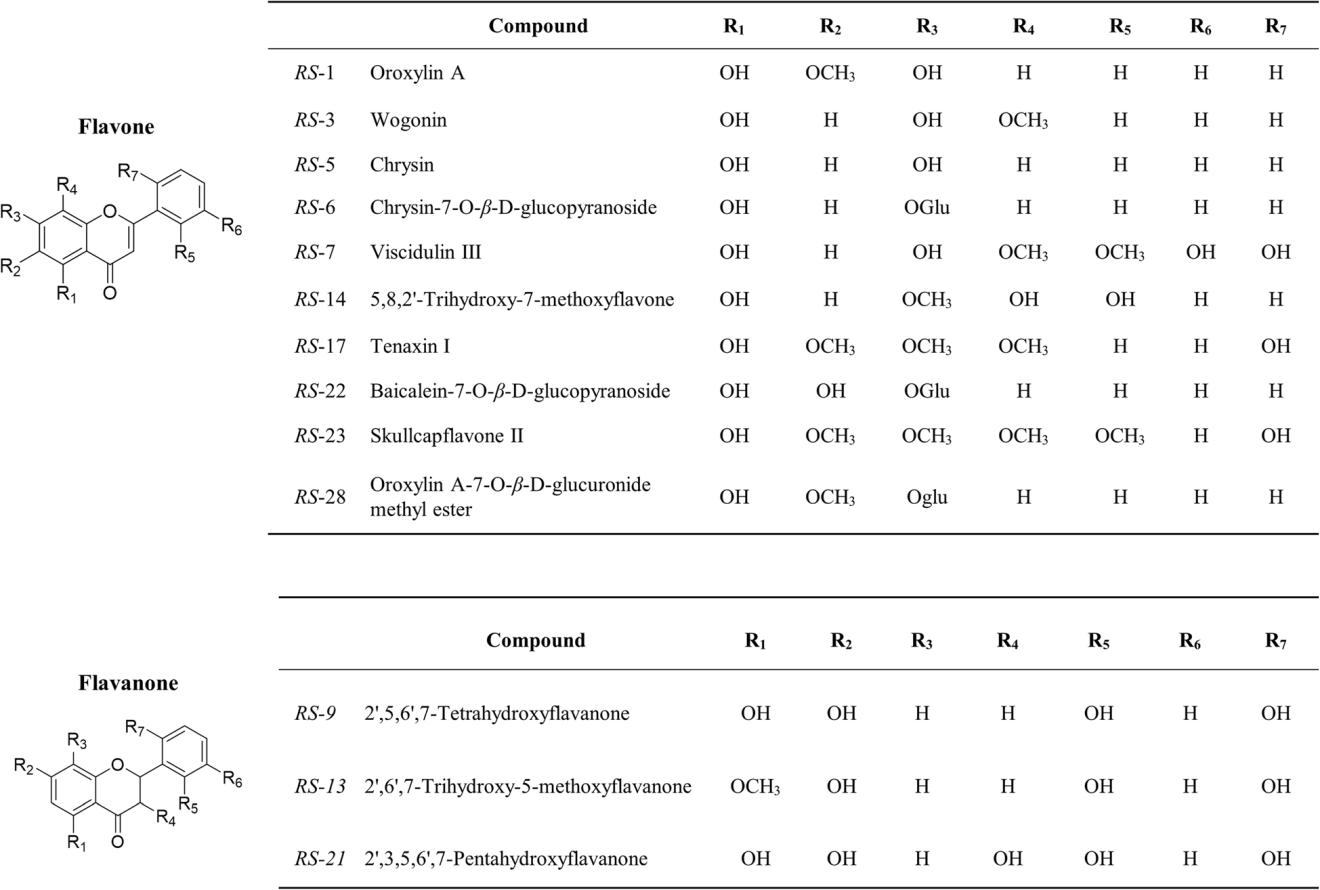
Chemical structures of the potential *α*-glucosidase inhibitors from flavonoids of *Radix Scutellariae* extract

Ultrafiltration LC-MS/MS has been widely used to screen bioactive compounds from natural products [55]. In this study, the complexes of *α*-glucosidase and ligands from *RS* were retained by an ultrafiltration membrane, whereas the unbound, low molecular weight compounds were washed away from the chamber. Subsequently, the remainings were dissociated, and the ligands were identified by LC-MS/MS. Finally, a total of 13 peaks were detected, yet not presented in control group, indicating a specific binding to *α*-glucosidase. Chemical structures of the trapped ligands are shown in Fig. 1, including wogonin, chrysin, oroxylin A, 5,8,2′-trihydroxy-7-methoxyflavone, and so on. These compounds were considered as potential *α*-glucosidase inhibitors, and reorganized as a chemical ingredients database for the next analysis.

Interactions between small molecules and proteins are often highly valued in biomedical and pharmaceutical sciences [56]. Numerous target proteins have been used for the treatment of T2D, such as insulin receptor, peroxisome proliferator activated receptor gamma (PPARG), and *α*-glucosidase [57]. In this study, target proteins of the 13 *α*-glucosidase inhibitors were collected using web tools. A total of 117 target proteins were obtained (Additional file 3). Some of them were therapeutic targets of T2D, such as bile acid receptor, histone deacetylase 1, prostaglandin G/H synthase 2, and so on [58]. It indicated that these *α***-**glucosidase inhibitors might affect T2D through multi-targets.

A pathway contains a panel of cascade reactions among various biomolecules [59]. Pathway analysis demonstrated that the 117 targets of *α*-glucosidase inhibitors were involved in 86 pathways (Additional file 3), including metabolism of xenobiotics by cytochrome P450, steroid hormone biosynthesis, insulin resistance, retinol metabolism, and so on. These data were further analyzed by network pharmacology.

### Compound-target-pathway (CTP) network and main active constituents of the *α*-glucosidase inhibitors from *RS*

**Fig. 2:**
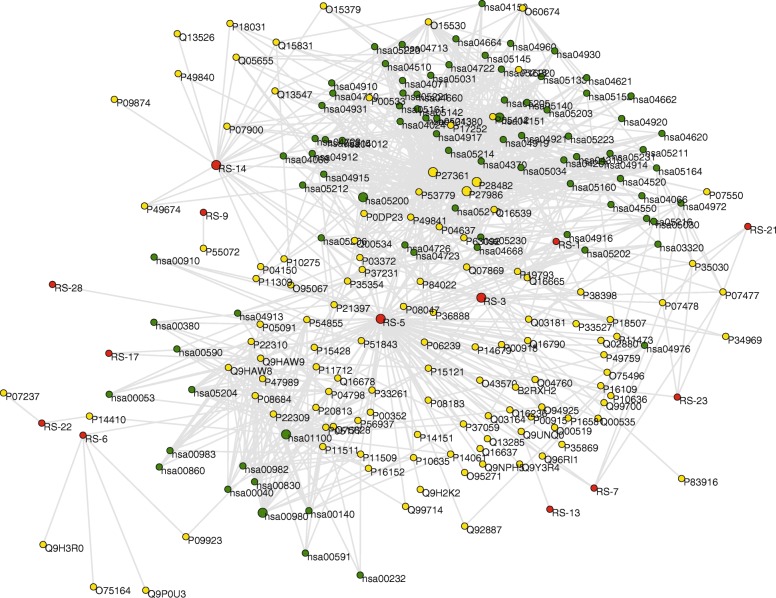
Compound-target-pathway (CTP) network of the potential *α*-glucosidase inhibitors from *RS*. The network consists of 13 red nodes (potential *α*-glucosidase inhibitors), 117 yellow nodes (target proteins), 86 green nodes (pathways), and 877 connections. The larger circles denote key nodes with the most connections. Node information is listed in Additional file 4. Gray lines represent connections

Natural products have advantages of multi-components and multi-targets [60]. Their pharmacological effects are the consequence of a series of interactional biochemical reactions. On the other hand, T2D is a chronic degenerative disease involving various genetic and environmental factors [61]. These elements cause great difficulty in the related researches. Network methodology provides us a great opportunity to solve the problem from a systemic perspective. In the present study, a compound-target-pathway network was first built for the *α*-glucosidase inhibitors, target proteins and related pathways (Fig. 2). This network contained 216 nodes (*N* = 216) and 877 connections (*E* = 877). The nodes consisted of 13 *α*-glucosidase inhibitors (red nodes), 117 target proteins (yellow nodes), and 86 pathways (green nodes). The larger circles denote the nodes with the most connections, and that gray lines represent connections. Annotations of these nodes were listed in Additional file 3.

**Fig. 3:**
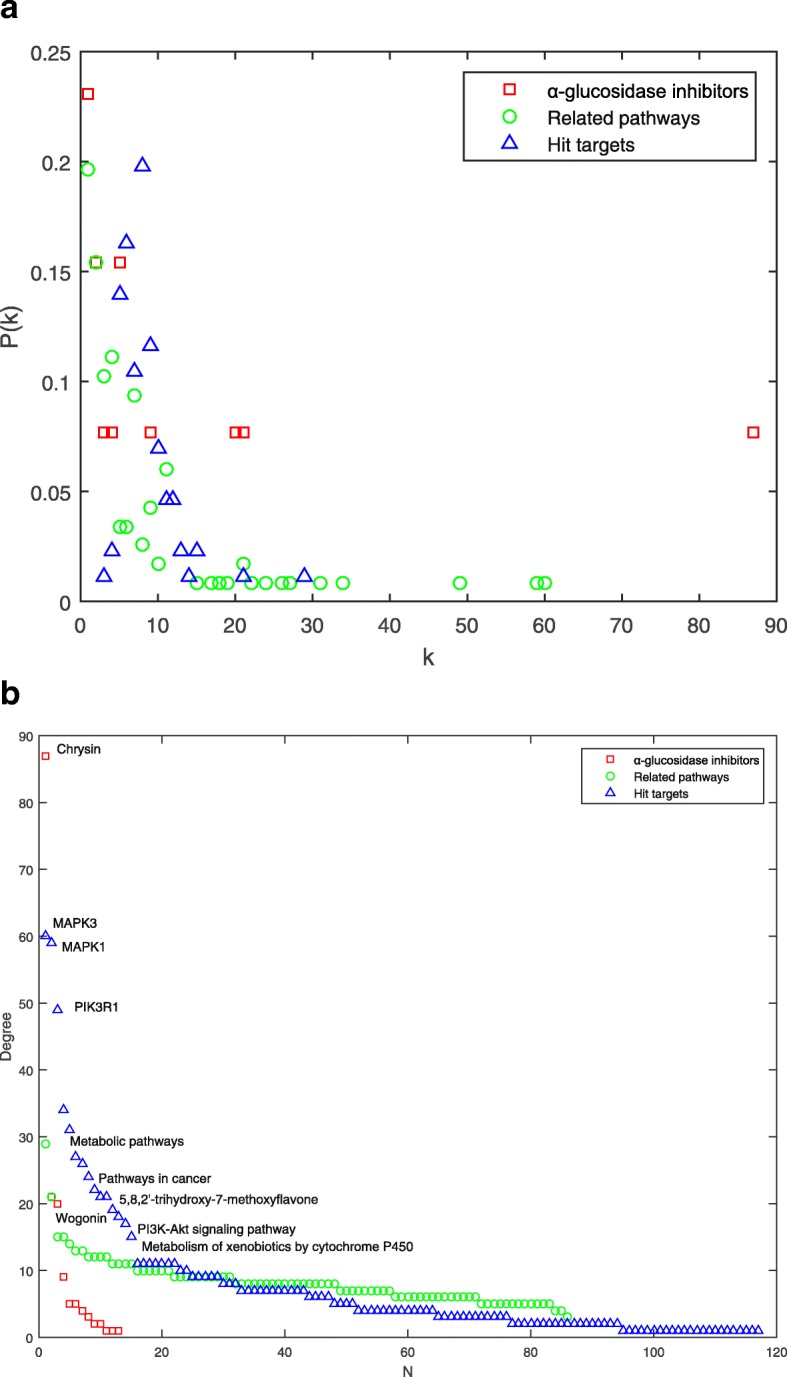
**a** Degree distribution of the CTP network. *k* represents degree values, and that *P(k)* indicates degree distribution. **b** Degree values (*k*) of all nodes in the CTP network, ranked in a descending order

Several topological parameters were calculated to describe characteristics of the network. Degree is the most elementary characteristic for a node, which tells us how many directly connected neighbors a node holds. ⟨*k*⟩ of the CTP network was 8.12, demonstrating that an average of more than eight neighbors were connected with one node. On the other hand, degree distribution measures the diversity of a network. Obviously, a few nodes had numerous neighbors, whereas others only had a small number of connections. Figure 3a shows that the red and blue nodes had an uneven distribution, whereas green nodes exhibited approximate scale-free property. The difference of *P(k)* for *α*-glucosidase inhibitors, hit targets and related pathways might be due to the complexity of natural products. The data suggested that a few highly connected nodes existed in the CTP network.

As interactions between molecules play a critical role in modulating the intrinsic biological processes, more attention should be paid to the highly-connected elements [62]. Within the framework of network science, the nodes with most connections are generally considered as hubs [63]. Although hubs are few in number, they are generally positioned to make strong contributions to global network function [63]. Disturbances on these hubs would spread rapidly throughout the entire network. Figure 3b shows a descending order of degree values for all nodes of the CTP network.

Among the *α*-glucosidase inhibitors, chrysin (*k* = 87) had the largest *k*, followed by 5,8,2′-trihydroxy-7-methoxyflavone (*k* = 21), and wogonin (*k* = 20). The three compounds exhibited much higher degree values than average (⟨*k*⟩ = 8.12). Chrysin treatment has been used to improve diabetes in rats, which could attenuate diabetes-induced impairment [64]. Wogonin could enhance the intracellular level of adiponectin, a therapeutic target for insulin resistance, diabetes, and diabetes-related complications [65]. Although there were few reports about bioactivity of 5,8,2′-trihydroxy-7-methoxyflavone, the activities of flavonoids are structure dependent with the hydroxylated phenolic structure [66], which should be tested in the future. These compounds have significant impacts on the global network function, and were considered as main active ingredients. They might contribute the most to the pharmacological effects of *α*-glucosidase inhibitors from *RS*.

Multi-targets could provide superior therapeutic effect and less side effect compared to a single action, especially in the treatment of complex diseases [67]. In the target proteins, mitogen-activated protein kinase 3 (MAPK3, *k* = 60), mitogen-activated protein kinase 1 (MAPK1, *k* = 59) and phosphoinositide-3-kinase regulatory subunit 1 (PIK3R1, *k* = 49) had much higher degree values than others. The two MAPKs have been found to be increased in human and rodent adipose tissue in the diabetic state [68]. PIK3R1 plays a key role in insulin signaling and diabetes [69]. The three proteins were interconnected with the most compounds and pathways in the CTP network, and were also considered as hub nodes.

Normal pathways maintain balance between complex intracellular and intercellular networks. The most highly connected pathway in the CTP network was metabolic pathways (hsa01100, *k* = 29), followed by pathways in cancer (hsa05200, *k* = 21), metabolism of xenobiotics by cytochrome P450 (hsa00980, *k* = 15) and PI3K-Akt signaling pathway (hsa04151, *k* = 15). Hsa01100 is a mega pathway defined in Kyoto Encyclopedia of Genes and Genomes (KEGG), that encompasses several other pathways [70], and was excluded to avoid redundant data. Hsa05200 is related to many diseases. A cross talk is existed between diabetes and obesity, and that diabetes has been shown to increase cancer risk [71]. Some drugs appear to reduce cancer incidence and improve prognosis of patients with diabetes [72]. Hsa00980 takes part in biotransformation of medicines in vivo [73]. Hsa04151 is required for insulin-dependent regulation on cellular metabolism, which was directly associated with T2D [74]. These reports indicated that the *α*-glucosidase inhibitors from *RS* might be work through various pathways.

### Protein-protein interaction (PPI) weighted network and key targets of the *α*-glucosidase inhibitors from *RS*

**Fig. 4:**
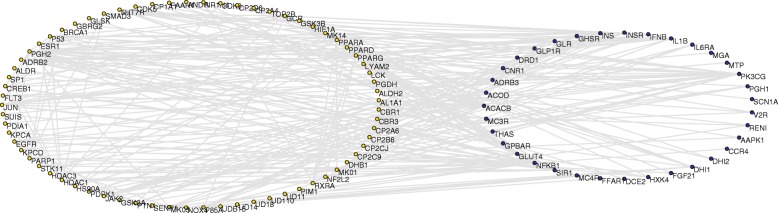
Protein-protein interaction (PPI) weighted network for the ligands of *α*-glucosidase inhibitors from *RS* and targets of T2D, containing 104 nodes and 228 connections. The yellow nodes are the targets of potential *α*-glucosidase inhibitors from *RS*, and that blue nodes represent therapeutic targets of T2D

Many biological systems found in biology contain numerous components, and the interactions between individual agents cause the emergence of structures and functions [75]. T2D is a typical polygenic disease affected by various therapeutic targets [76]. Elucidation of the interactions between targets of T2D and ligands of *α*-glucosidase inhibitors from *RS* would help to understand the molecular mechanisms. In this study, a total of 64 targets of T2D were collected, including 34 successful and 30 clinical trial targets. Coincidentally, nine of them were also the targets of *α*-glucosidase inhibitors from *RS*, such as bile acid receptor (NR1H4), histone deacetylase 1 (HDAC1), prostaglandin G/H synthase 2 (PGH2), and so on. We analyzed the functional associations between the two groups of proteins using STRING database. After preliminary exclusion of isolated nodes, 69 ligands of *α*-glucosidase inhibitors from *RS* and 35 drug-targets of T2D were preserved. A protein-protein interaction (PPI) weighted network was then constructed (Fig. 4) for the two groups of proteins, containing 104 nodes and 228 connections.

**Fig. 5:**
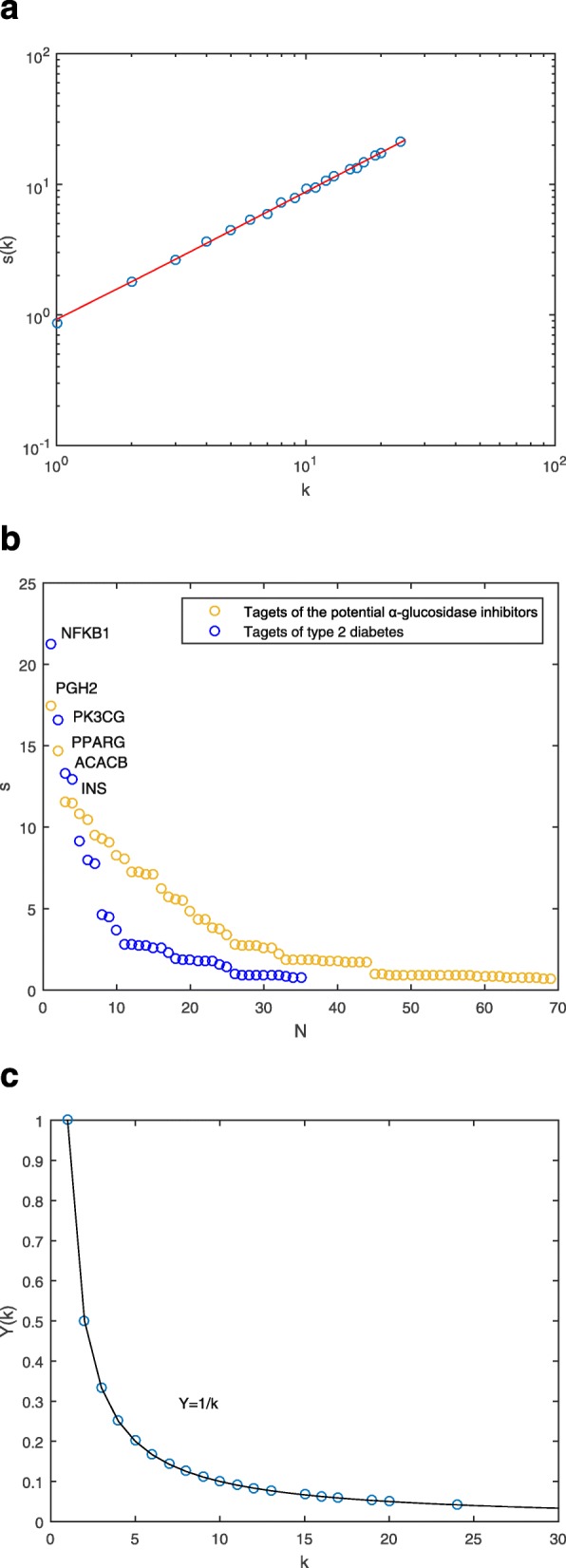
**a** Average strength *s(k)* as a function of degree *k* in logarithmic coordinates. The data points are fitted to a straight line, showing the relation *s(k) ∼ k*^*β*^*.***b** Node strengths of the PPI network sorted in descending order. **c** Disparity *Y(k)* in the edge weight decays as a function of *k*. The data points are well approximated by the curve *Y(k)* = 1/*k*

In this PPI weighted network, degree *k*_*i*_ represents the number of other proteins interacted with node *i*. Node strength (*s*_*i*_) describes the interactive intensities between the two groups of proteins. This parameter comprehensively reflects local information of node *i* by considering neighbor nodes and edge weights. Correlation between degree *k* and the average strength *(k)* for nodes with specific *k* values were investigated. If *s(k) ∼ k*^*β*^ with an exponent *β ≠* 1, the edge weight is correlated with the network topology. Figure 5a depicts the correlation between *k* and *s(k)* for the PPI network*.* The *s(k)* increased with *k* as *s(k) ∼ k*^*β*^ with the exponent *β ≈* 0.87, indicating that the node strengths were closely associated with degrees.

Node strengths of the PPI network were then sorted in descending order (Fig. 5b). Larger strengths point to nodes with larger degrees. Nuclear factor kappa B subunit 1 (NFKB1) had the strongest interactions (*s* = 21.26) with the targets of the potential *α*-glucosidase inhibitors, followed by phosphatidylinositol-4,5-bisphosphate 3-kinase catalytic subunit gamma (PK3CG, *s* = 16.61), acetyl-CoA carboxylase beta (ACACB, *s* = 13.32), and insulin precursor (INS, *s* = 12.96). In the targets of the potential *α*-glucosidase inhibitors, PGH2 (*s* = 17.47) and PPARG (*s* = 14.66) interacted with the most nodes of T2D. Moreover, the two proteins were also successful targets of T2D. These nodes occupied important positions in the overall organization of PPI network.

System behaviors are dependent largely on the overall structure rather than the individual parts. Disparity *Yi* depicts the dispersion of weight distribution [77]. If the weights of all edges are approximately equal, Y_*i*_ ∝ 1/*k*_*i*_. If one edge weight is important whereas the others negligible, *Y*_*i*_ ≈ 1. It is obvious that *Yi* is associated with *k*_*i*_. *Y(k)* often attracts more attentions in the weighted network. It is the average of *Y*_*i*_ for all nodes with degree *k*. If the weight distribution is relatively uniform, *Y(k)* ∝ 1/*k*, otherwise *Y(k)* ≈ 1. As shown in Fig. 5c, the average disparity *Y(k)* ∝ 1/*k* in the PPI network. It demonstrated that the edge weight distribution for nodes with the same degree *k* was approximately equal.

**Fig. 6:**
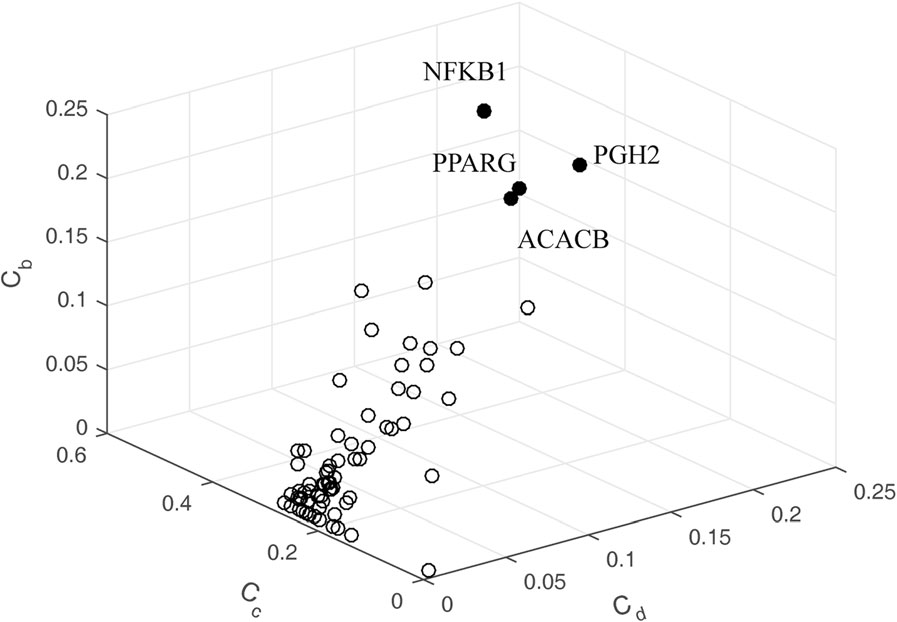
Three-dimensional diagram of degree centrality (*C*_*d*_), betweenness centrality (*C*_*b*_) and closeness centrality (*C*_*c*_) for the nodes in PPI network

Changes in central positions of the network are always more important than those in marginal or relatively isolated positions [78]. To determine central nodes of the PPI network, three centrality indices, degree centrality, betweenness centrality, and closeness centrality were integrated into a three-dimensional diagram (Fig. 6). Distribution of these values seemed roughly uniform. However, a few nodes appeared as outliers. PPARG (*C*_*d*_ = 0.165, *C*_*b*_ = 0.232, *C*_*c*_ = 0.401), ACACB (*C*_*d*_ = 0.155, *C*_*b*_ = 0.184, *C*_*c*_ = 0.318), NFKB1 (*C*_*d*_ = 0.233, *C*_*b*_ = 0.161, *C*_*c*_ = 0.431), and PGH2 (*C*_*d*_ = 0.194, *C*_*b*_ = 0.157, *C*_*c*_ = 0.427) showed higher centrality scores than other nodes. A total of 54 neighbors were found to be connected with these central nodes, accounting for 51.9% of the total target proteins. The highly connectivity of the four proteins indicated that they could affect the PPI weighted network greatly.

PPARG and PGH2 are both important targets of the *α*-glucosidase inhibitors and T2D. Recent studies have demonstrated the association of PPARG with T2D. PPARG is a master transcriptional regulator of adipocyte differentiation. Variants in PPARG with decreased activity in adipocyte differentiation were found to be associated with increased risk of T2D [79]. A family-based study of Mexican Americans showed that variation in PPARG contributes to declining insulin resistance and concomitant deterioration in *β*-cell function at risk for T2D [80]. PGH2 generates prostaglandins and causes insulin insensitivity. PGH2 polymorphisms were found to be associated with T2D in Pima Indians comprising 1000 subjects [81]. Another variant of PGH2 had a protective role against T2D in two German cohorts [82].

ACACB and NFKB1 are therapeutic targets of T2D, and have a strong relationship with targets of *α*-glucosidase inhibitors from *RS*. ACACB is a regulator of fatty acid metabolism. It catalyzes the carboxylation of acetyl-CoA to malonyl-CoA. The problems in fatty acid metabolism can lead to insulin resistance, which is a precursor for T2D. Polymorphisms of ACACB are associated with T2D in postmenopausal women and Pakistani Punjabis [83, 84]. NFKB1 encodes a subunit of NF-kappa B. It is specifically involved in anti-inflammatory effects, and that inflammation is linked to insulin resistance and diabetes. Two common NFKB1 variants were found to be involved in T2D in an elderly cohort [85]. A transcriptome and proteome study demonstrated that NFKB1 were increased in expression in diabetic subjects [86]. These reports further confirmed the importance of PPARG, PGH2, ACACB, and NFKB1 to the *α***-**glucosidase inhibitors from *RS*.

### Core subnetwork and critical pathways of the *α*-glucosidase inhibitors from *RS*

**Fig. 7:**
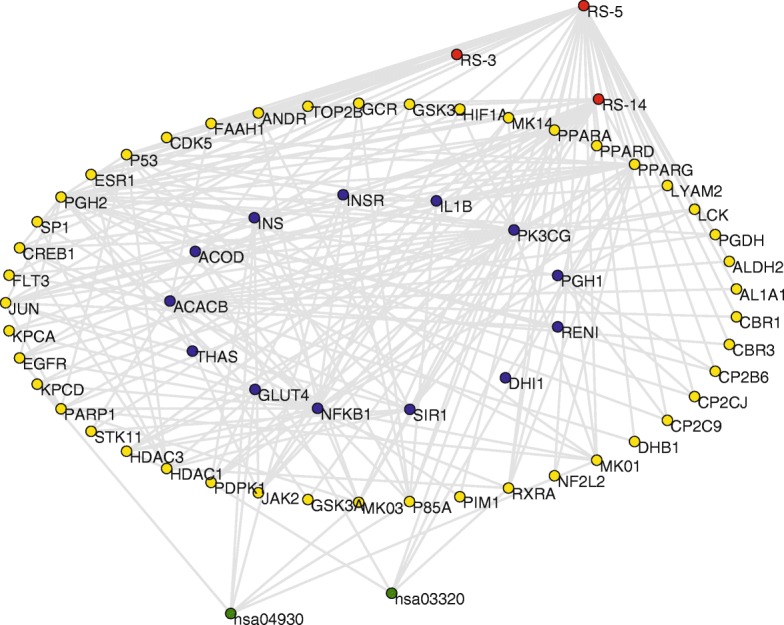
Core subnetwork for the potential *α*-glucosidase inhibitors and type 2 diabetes mellitus, consisted of 29 nodes and 47 connections. The yellow nodes are the targets of potential *α*-glucosidase inhibitors from *RS*, and that blue nodes indicate therapeutic targets of T2D. Red and green nodes represent the related *α*-glucosidase inhibitors and pathways, respectively

In order to get further understanding of the key targets, the nodes connected to PPARG, ACACB, NFKB1, and PGH2 were extracted from PPI network. A total of 45 targets of the *α*-glucosidase inhibitors and 13 drug targets of T2D were selected. Pathway analysis indicated that these proteins were involved in 91 pathways. After querying KEGG DISEASE database, the type II diabetes mellitus pathway (hsa04930) and PPAR signaling pathway (hsa03320) showed a direct correlation with T2D. Therefore, the two processes might play significant roles in the pharmacological activities of the *α*-glucosidase inhibitors from *RS*. Moreover, we also extracted the *α*-glucosidase inhibitors connected to these key targets from CTP network, including chrysin, 5,8,2′-trihydroxy-7-methoxyflavone, and wogonin. All these elements were reorganized as a core subnetwork (Fig. 7), consisted of 63 nodes and 220 connections.

**Fig. 8:**
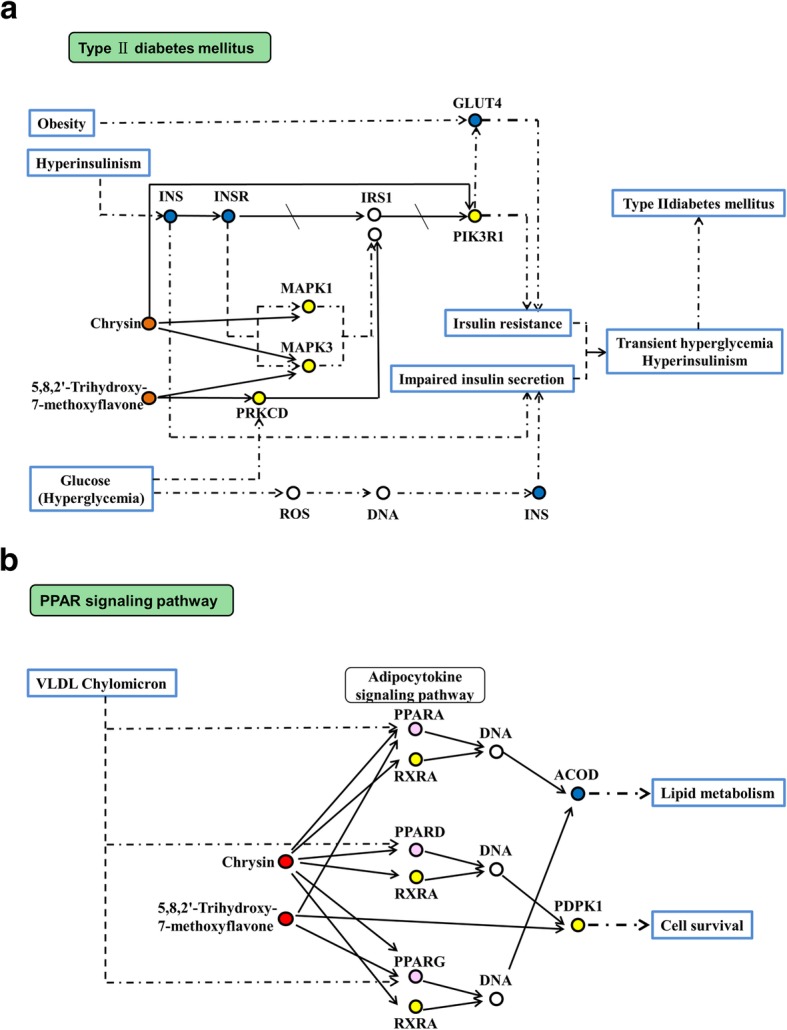
Critical pathways of the potential *α*-glucosidase inhibitors from *RS*. **a** Type 2 diabetes mellitus pathway. **b** PPAR signaling pathway. The yellow nodes are the targets of potential *α*-glucosidase inhibitors from *RS*, blue nodes indicate therapeutic targets of T2D, and that pink nodes denote targets belonged to both the two groups. Red nodes represent the related *α*-glucosidase inhibitors

In this core subnetwork, chrysin, 5,8,2′-trihydroxy-7-methoxyflavone, and seven targets, MAPK1, MAPK3, PIK3R1, protein kinase C delta (PRKCD), INS, insulin receptor (INSR), solute carrier family 2 member 4 (GLUT4) belonged to hsa04930. As Fig. 8a shown, type II diabetes mellitus contains various kinases. MAPK1 and MAPK3 (also known as ERK2 and ERK1) play an important role in the MAPK/ERK cascade. Diabetogenic factors have been found to affect insulin signaling through activation of the ERK signaling pathway [87]. Previous research has revealed that targeting of the ERK pathway held promise for the treatment of T2D [88]. In the present study, MAPK1 and MAPK3 were the targets of chrysin and 5,8,2′-trihydroxy-7-methoxyflavone. In addition, PIK3R1 is necessary for the insulin-stimulated increase in glucose uptake and glycogen synthesis in insulin-sensitive tissues. Mutations of PIK3R1 could cause insulin resistance, which is strongly associated with insulin resistance [69]. Interestingly, chrysin, 5,8,2′-trihydroxy-7-methoxyflavone, MAPK1, MAPK3 and PIK3R1 were also hub nodes of the CTP network.

Chrysin, 5,8,2′-trihydroxy-7-methoxyflavone, and another six targets, peroxisome proliferator activated receptor alpha (PPARA), peroxisome proliferator activated receptor delta (PPARD), PPARG, retinoid X receptor alpha (RXRA), 3-phosphoinositide dependent protein kinase 1 (PDPK1), stearoyl-CoA desaturase (ACOD) were involved in hsa03320 (Fig. 8b). Accumulating evidence highlighted the role of PPAR signaling in T2D [89]. PPARs (Peroxisome proliferator-activated receptors) are nuclear hormone receptors that are activated by fatty acids and their derivatives, containing three subtypes (PPAR alpha, beta/delta, and gamma). The three PPAR isoforms all appeared in hsa03320. They were both the targets of the *α*-glucosidase inhibitors from *RS* and T2D. Moreover, PPARG was the central node of PPI weight network. It could promote adipocyte differentiation to enhance blood glucose uptake. In hsa03320 process, chrysin and 5,8,2′-trihydroxy-7-methoxyflavone were directly connected with the PPARs. This further confirmed the importance of the two *α*-glucosidase inhibitors. These data supported the hypothesis that the *α*-glucosidase inhibitors from *RS* might influence T2D through hsa04930 and hsa03320 processes.

## Discussion

Many previous studies have investigated the antidiabetic constituents of *Radix scutellariae*. For instance, Cui et al. used a tandem quadrupole mass spectrometer coupled with enzyme activity analysis to explore hypoglycemic effect of *RS* and *Coptidis rhizome* [90]. This method was accurate and sensitive enough for quantitative evaluation of seven major components and six enzymes. The results indicated that combined extract had stronger effects on T2D through multiple components against multiple targets. Tahtah et al. applied triple aldose reductase/*α*-glucosidase/radical scavenging high-resolution profiling combined with high-performance liquid chromatography-high-resolution mass spectrometry-solid-phase extraction-nuclear magnetic resonance spectroscopy to identify antidiabetic constituents from *RS* [91]. Baicalein was screened as *α*-glucosidase inhibitor. In another approach, a systematic study on metabolism and activity evaluation of *Radix Scutellaria* extract in rat plasma was conducted, using UHPLC with quadrupole time-of-flight mass spectrometry [92]. Wogonoside, norwogoin-7-O-Glu acid and oroxyloside exhibited better binding affinities with *α*-glucosidase. These results are partially consistent with those obtained in our study. While they demonstrate versatility and success of phytochemical analysis in the identification of novel ligands for therapeutic targets, these studies are labor-intensive and time consuming [55]. Moreover, the mechanism analysis of active constituents is often limited by existing knowledges and experiences. Our study constructs a network model of compounds, target proteins and pathways to explore mechanism of *α*-glucosidase inhibitors identified by ultrafiltration LC-MS from *RS*. This approach is more rapidly and extensive as the application of computational tools as well as systems biology. The selected main active components, key targets and critical pathways would provide more information for the interpretation of *Radix Scutellaria* and T2D. However, these results are mainly based on statistical analysis and prediction. Further studies on cell and animal models are required to give a distinct answer.

## Conclusion

This study presents one of the first systematic analyses of *α*-glucosidase inhibitors from natural products using ultrafiltration LC-MS/MS and network pharmacology. Our findings suggested a possible application of flavonoids from *Radix scutellariae* in the treatment of T2D. The *n*-butanol part of ethanol extract from *RS* showed strong *α*-glucosidase inhibition activity. Thirty-two kinds of flavonoids were identified from the extract, and 13 of them were screened as *α*-glucosidase inhibitors, including viscidulin III, oroxylin A, 2′,3,5,6′,7-pentahydroxyflavanone, and so on. The results were strongly supported by previous reports about natural flavonoids and T2D [4, 5, 14, 22, 66]. These compounds, together with their target proteins and related pathways, were integrated into three complex networks. The underlying mechanism of these natural *α*-glucosidase inhibitors were revealed by network analyses. Chrysin, 5,8,2′-trihydroxy-7-methoxyflavone and wogonin were the main active constituents. PPARG, PGH2, ACACB, and NFKB1 were key targets. The α-glucosidase inhibitors from *RS* might influence T2D progression through the type II diabetes mellitus and PPAR signaling pathways. In all, the combination of ultrafiltration LC-MS and system pharmacology would enable to generate novel insight into the research of plant medicines.

## Supplementary information

**Additional file 1. **Total ions chromatogram (TIC) of the *n*-butanol part of ethanol extract from *Radix Scutellariae* by UPLC-TripleTOF.

**Additional file 2. **Primary fragmentation pathways of compound 14 (oroxylin A-7-O-*β*-D-Glucuronopyranoside), 16 (wogonoside), 17 (4′-hydroxywogonin), 23 (5,8,2′-trihydroxy-7-methoxyflavone), 26 (wogonin) and 28 (chrysin).

**Additional file 3. ***α*-glucosidase inhibition curves of crude extract of *Radix scutellariae* and acarbose.

**Additional file 4.** Information of nodes in the compound-target-pathway (CTP) network.

**Additional file 5.** Degree values of CTP network, node strength and centrality indices of PPI network.

## Data Availability

Datasets supporting the results of this article have been included in the additional files.

## Abbreviations

ACACB: Acetyl-CoA carboxylase beta
ACOD: Stearoyl-CoA desaturase
CI: Centrality indices
CTP: Compound-target-pathway
GLUT4: Solute carrier family 2 member 4
HDAC1: Histone deacetylase 1
hsa00980: Metabolism of xenobiotics by cytochrome P450
hsa01100: Metabolic pathways
hsa03320: PPAR signaling pathway
hsa04151: PI3K-Akt signaling pathway
hsa04930: Type II diabetes mellitus pathway
INS: Insulin precursor
INSR: Insulin receptor
KEGG: Kyoto Encyclopedia of Genes and Genomes
MAPK1: Mitogen-activated protein kinase 1
MAPK3: Mitogen-activated protein kinase 3
NFKB1: Nuclear factor kappa B subunit 1
NR1H4: Bile acid receptor
PDPK1: 3-phosphoinositide dependent protein kinase 1
PGH2: Prostaglandin G/H synthase 2
PIK3R1: Phosphoinositide-3-kinase regulatory subunit 1
PK3CG: Phosphatidylinositol-4,5-bisphosphate 3-kinase catalytic subunit gamma
PPARA: Peroxisome proliferator activated receptor alpha
PPARD: Peroxisome proliferator activated receptor delta
PPARG: Peroxisome proliferator activated receptor gamma
PPI: Protein-protein interaction
PRKCD: Protein kinase C delta
*RS*: *Radix scutellariae*
RXRA: Retinoid X receptor alpha
STRING: Search Tool for the Retrieval of Interacting Genes/Proteins
T2D: Type 2 diabetes
Triple TOF-MS: Triple time-of-flight mass spectrometer
UPLC: Ultra high performance liquid chromatography
