# Supplementary material for: A systematic analysis of natural α-glucosidase inhibitors from flavonoids of Radix scutellariae using ultrafiltration UPLC-TripleTOF-MS/MS and network pharmacology
Source: BMC Complement Med Ther. 2020 Mar 6;20:72. doi: 10.1186/s12906-020-2871-3 (PMC7076893; doi:10.1186/s12906-020-2871-3)

Additional file 1 Total ions chromatogram (TIC) of the *n*-butanol part of ethanol extract from *Radix Scutellariae* by UPLC-TripleTOF

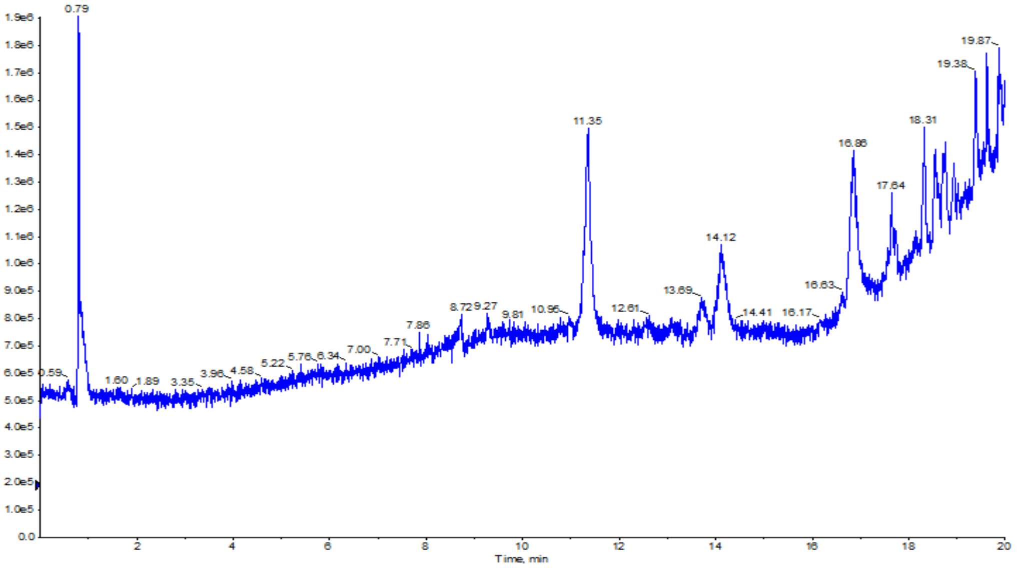

Supplement: Supplementary file 1 — Additional file 1. Total ions chromatogram (TIC) of the n-butanol part of ethanol extract from Radix Scutellariae by UPLC-TripleTOF. [file 12906_2020_2871_MOESM1_ESM.pdf]
