# Supplementary material for: A systematic analysis of natural α-glucosidase inhibitors from flavonoids of Radix scutellariae using ultrafiltration UPLC-TripleTOF-MS/MS and network pharmacology
Source: BMC Complement Med Ther. 2020 Mar 6;20:72. doi: 10.1186/s12906-020-2871-3 (PMC7076893; doi:10.1186/s12906-020-2871-3)

Additional file 2 Primary fragmentation pathways of compound 14 (oroxylin A-7-O-*β*-D-Glucuronopyranoside), 16 (wogonoside), 17 (4'-hydroxywogonin), 23 (5,8,2'-trihydroxy-7-methoxyflavone), 26 (wogonin) and 28 (chrysin)

(a) Compound 28: chrysin

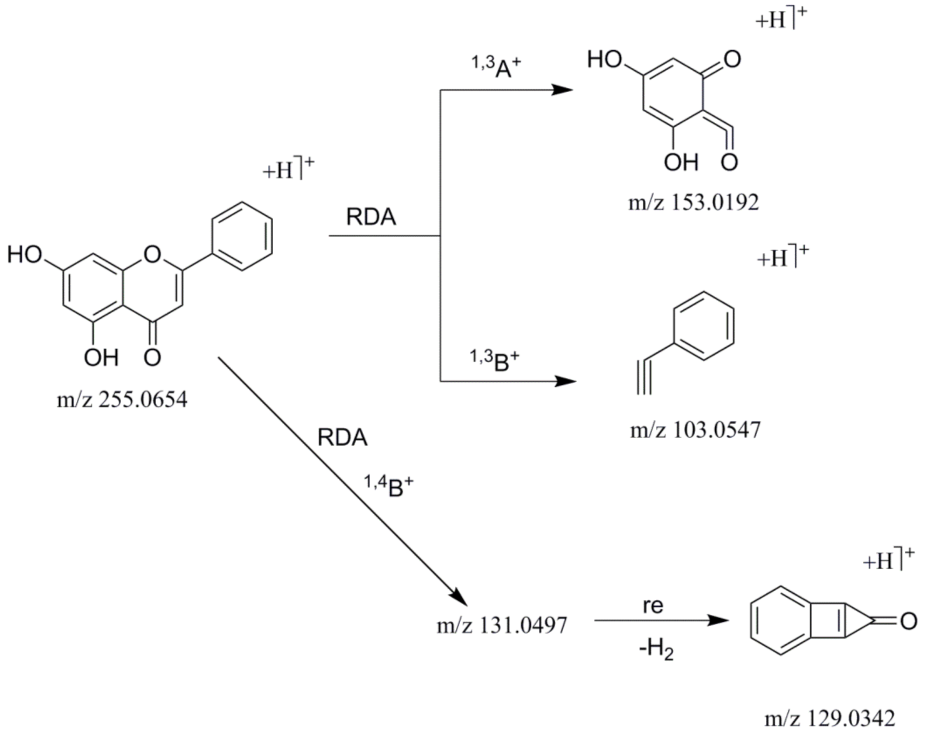

(b) Compound 26: wogonin

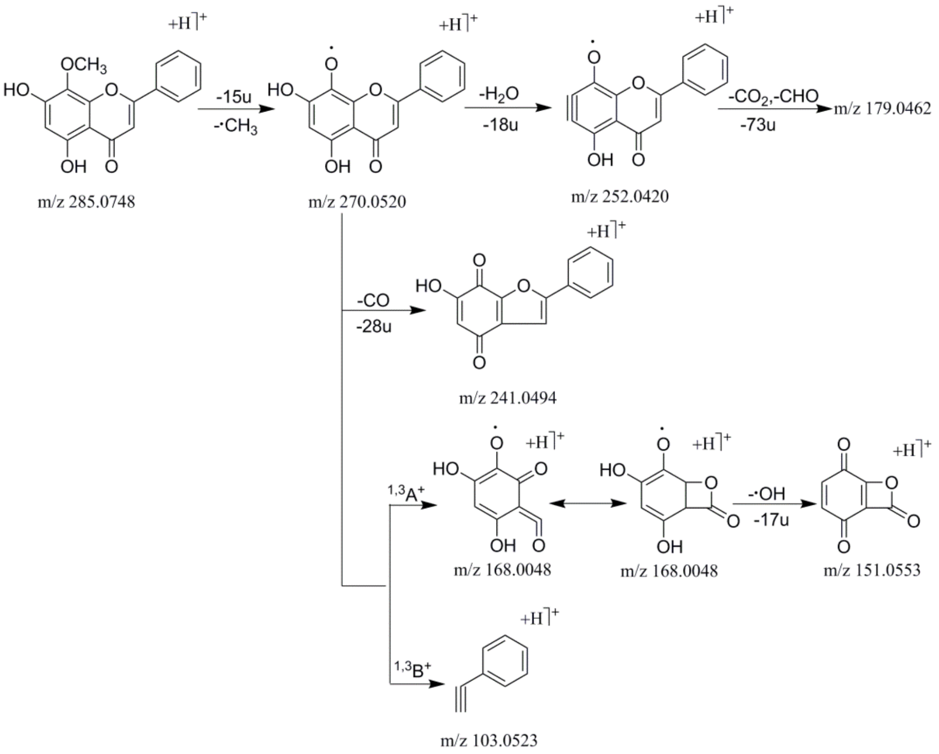

(c) Compound 17: 4'-hydroxywogonin

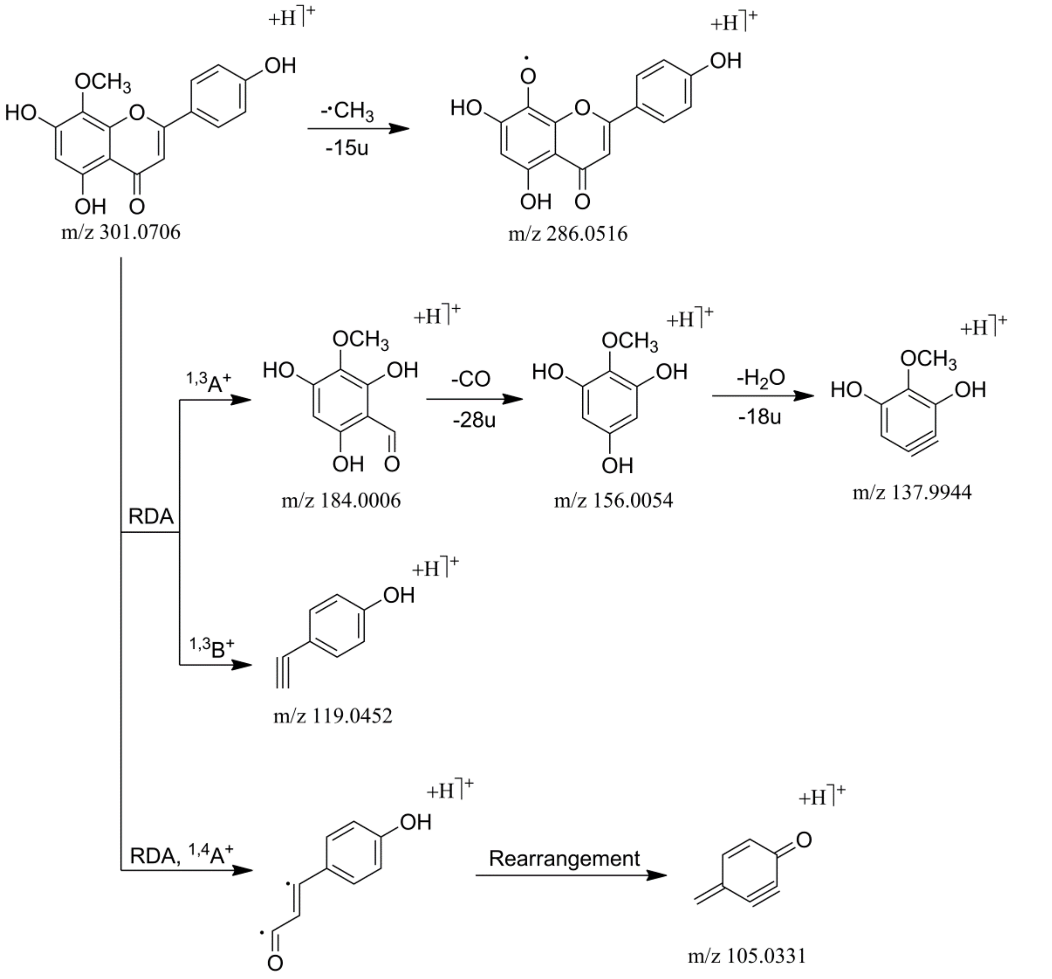

(d) Compound 23: 5,8,2'-trihydroxy-7-methoxyflavone

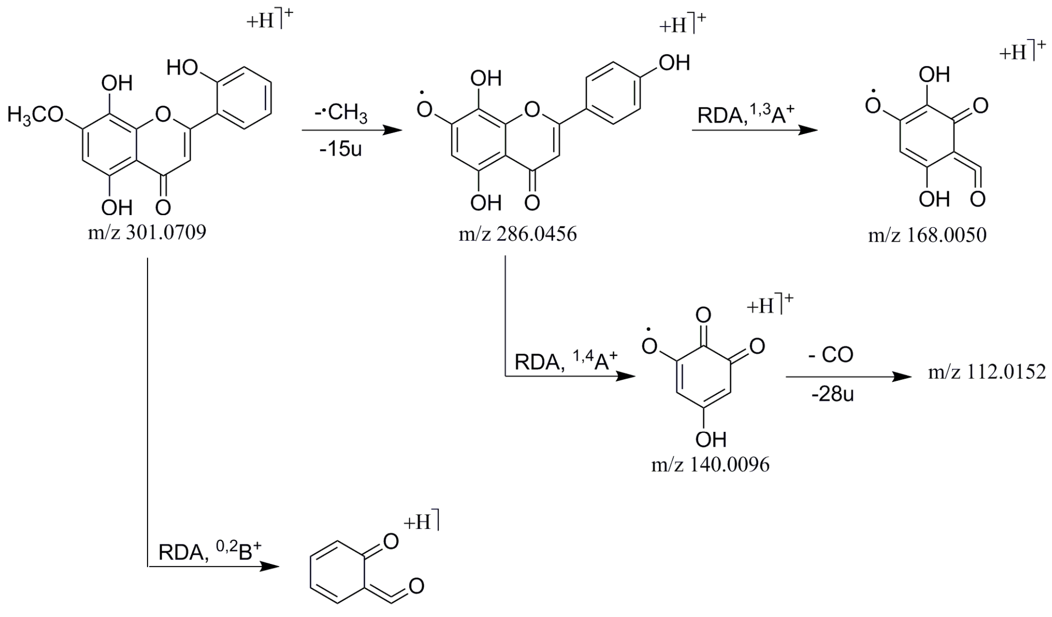

(e) Compound 14: Oroxylin A-7-O-β-D-glucuronopyranoside

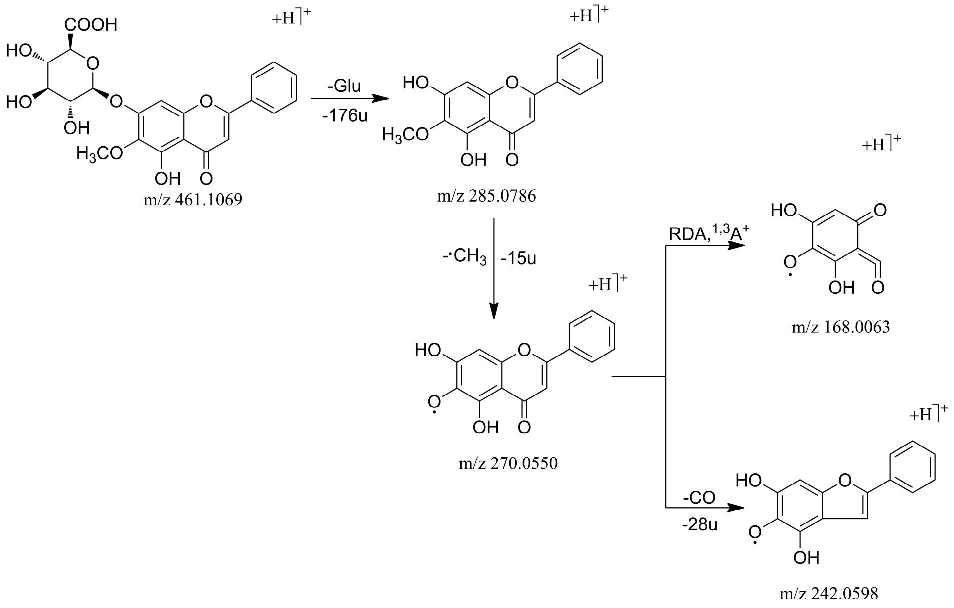

(f) Compound 16: Wogonoside

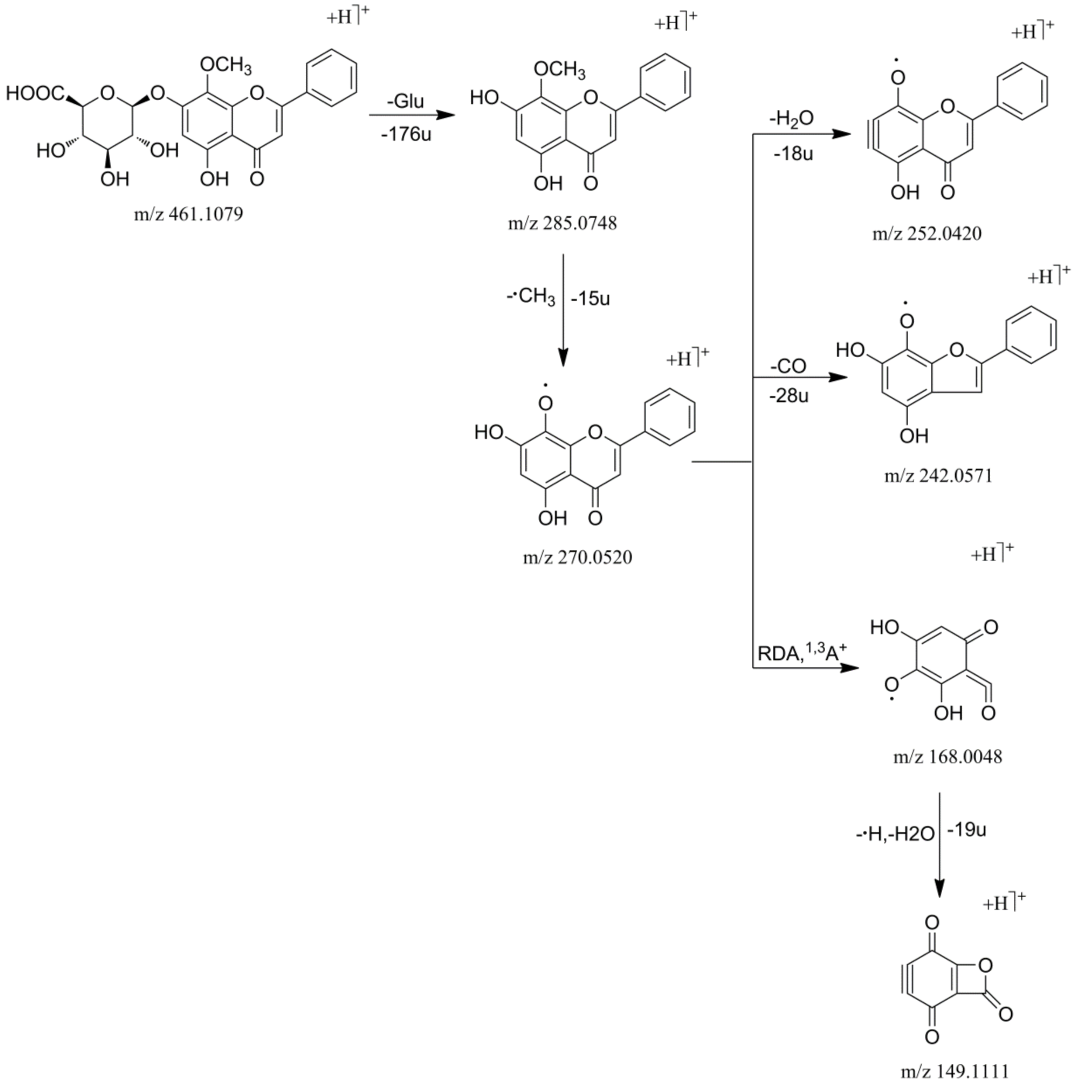

Supplement: Supplementary file 2 — Additional file 2. Primary fragmentation pathways of compound 14 (oroxylin A-7-O-β-D-Glucuronopyranoside), 16 (wogonoside), 17 (4′-hydroxywogonin), 23 (5,8,2′-trihydroxy-7-methoxyflavone), 26 (wogonin) and 28 (chrysin). [file 12906_2020_2871_MOESM2_ESM.pdf]
