# Supplementary material for: A systematic analysis of natural α-glucosidase inhibitors from flavonoids of Radix scutellariae using ultrafiltration UPLC-TripleTOF-MS/MS and network pharmacology
Source: BMC Complement Med Ther. 2020 Mar 6;20:72. doi: 10.1186/s12906-020-2871-3 (PMC7076893; doi:10.1186/s12906-020-2871-3)

**Additional file 3** *α*-glucosidase inhibition curves of crude extract of *Radix scutellariae* **(a)**, and acarbose **(b)**

**(a)**


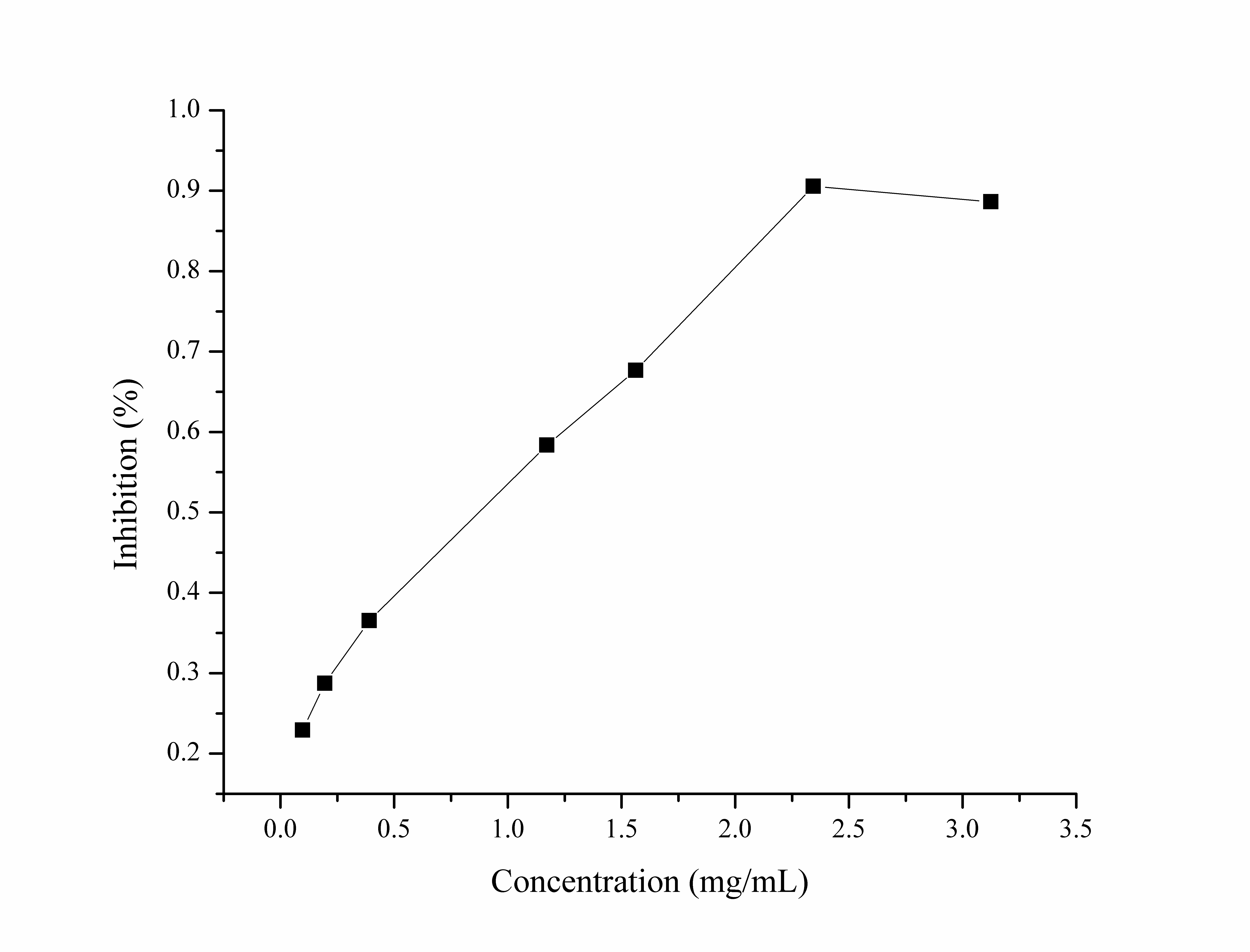


**(b)**


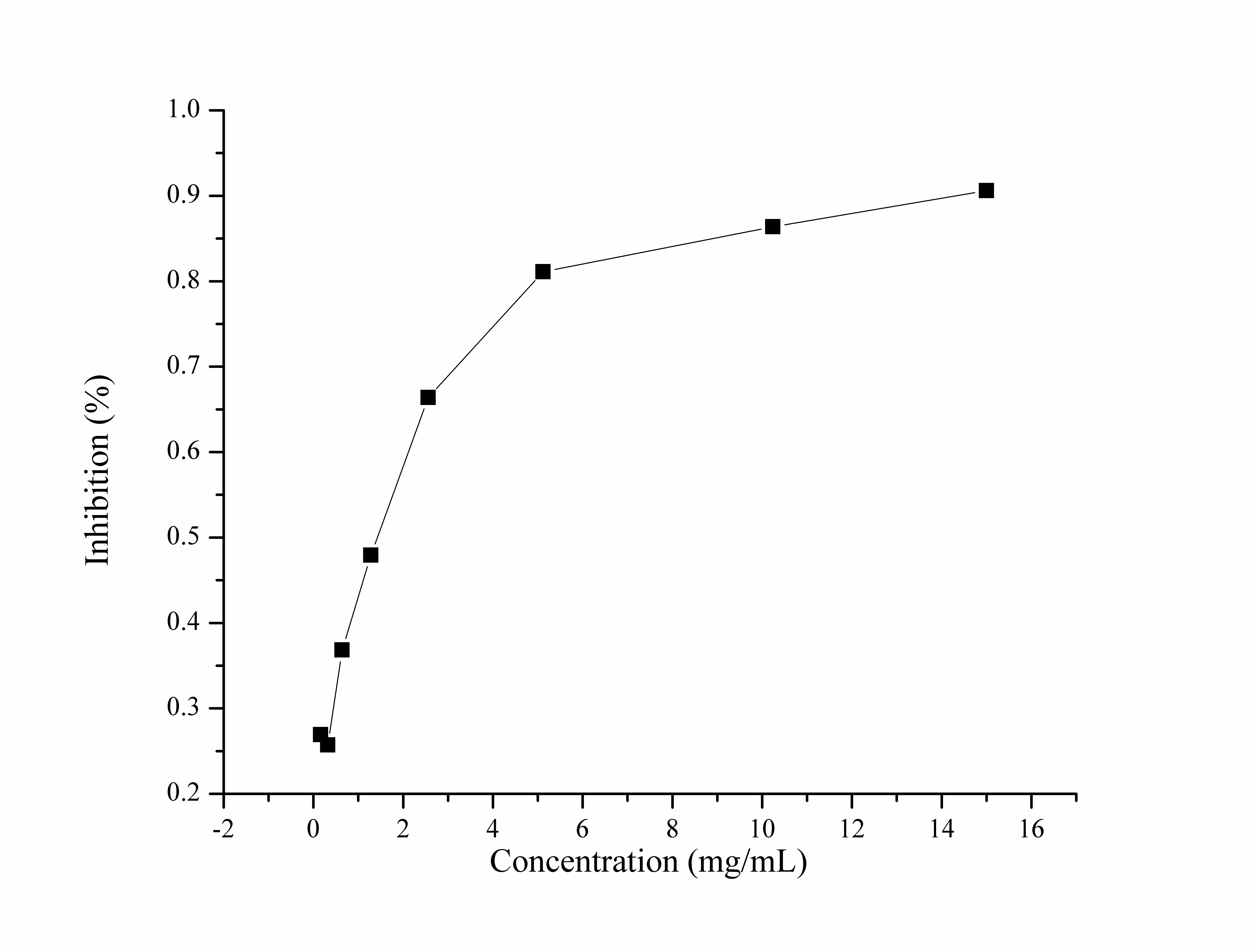

Supplement: Supplementary file 3 — Additional file 3. α-glucosidase inhibition curves of crude extract of Radix scutellariae and acarbose. [file 12906_2020_2871_MOESM3_ESM.docx]
