# Supplementary material for: A systematic analysis of natural α-glucosidase inhibitors from flavonoids of Radix scutellariae using ultrafiltration UPLC-TripleTOF-MS/MS and network pharmacology
Source: BMC Complement Med Ther. 2020 Mar 6;20:72. doi: 10.1186/s12906-020-2871-3 (PMC7076893; doi:10.1186/s12906-020-2871-3)
Supplement: Supplementary file 4 — Additional file 4. Information of nodes in the compound-target-pathway (CTP) network. [file 12906_2020_2871_MOESM4_ESM.docx]

**Additional file 4** Information of nodes in the compound-target-pathway (CTP) network

| **No.** | **Compound** ^a^ | **No.** | **Target** ^b^ | **No.** | **Pathway** ^c^ |
| --- | --- | --- | --- | --- | --- |
| 1 | Oroxylin A | 14 | P05412 | 131 | hsa00980 |
| 2 | Wogonin | 15 | P36888 | 132 | hsa00140 |
| 3 | Chrysin | 16 | P16220 | 133 | hsa05204 |
| 4 | Chrysin-7-O-*β*-D-glucopyranoside | 17 | P08047 | 134 | hsa00982 |
| 5 | Viscidulin III | 18 | P51843 | 135 | hsa00830 |
| 6 | 2',5,6',7-tetrahydroxyflavane | 19 | P15121 | 136 | hsa04915 |
| 7 | 2',6',7-trihydroxy-5-methoxyflavanone | 20 | P07477 | 137 | hsa04919 |
| 8 | 5,8,2'-trihydroxy-7-methoxyflavone | 21 | P35030 | 138 | hsa05200 |
| 9 | Tenaxin I | 22 | P07478 | 139 | hsa05223 |
| 10 | 2',3,5,6',7-pentahydroxyflavanone | 23 | P07550 | 140 | hsa00983 |
| 11 | Baicalein-7-O-*β*-D-glucopyranoside | 24 | P35354 | 141 | hsa05215 |
| 12 | Skullcapflavone II | 25 | P03372 | 142 | hsa04913 |
| 13 | Oroxylin A 7-O-*β*-D-glucuronide methyl ester | 26 | P04637 | 143 | hsa04912 |
|  |  | 27 | P38398 | 144 | hsa04722 |
|  |  | 28 | P83916 | 145 | hsa00040 |
|  |  | 29 | P18507 | 146 | hsa05231 |
|  |  | 30 | O75496 | 147 | hsa05160 |
|  |  | 31 | O94925 | 148 | hsa04068 |
|  |  | 32 | P84022 | 149 | hsa05210 |
|  |  | 33 | P34969 | 150 | hsa04726 |
|  |  | 34 | P49759 | 151 | hsa05230 |
|  |  | 35 | Q00535 | 152 | hsa00053 |
|  |  | 36 | P04798 | 153 | hsa05214 |
|  |  | 37 | O00519 | 154 | hsa05212 |
|  |  | 38 | P10275 | 155 | hsa05164 |
|  |  | 39 | Q96RI1 | 156 | hsa04917 |
|  |  | 40 | P00918 | 157 | hsa05213 |
|  |  | 41 | Q00534 | 158 | hsa04668 |
|  |  | 42 | P11511 | 159 | hsa04012 |
|  |  | 43 | P10635 | 160 | hsa04071 |
|  |  | 44 | P08684 | 161 | hsa04664 |
|  |  | 45 | Q02880 | 162 | hsa04151 |
|  |  | 46 | P04150 | 163 | hsa04976 |
|  |  | 47 | P49841 | 164 | hsa04728 |
|  |  | 48 | Q16665 | 165 | hsa04916 |
|  |  | 49 | Q16539 | 166 | hsa04380 |
|  |  | 50 | P21397 | 167 | hsa04723 |
|  |  | 51 | P33527 | 168 | hsa04660 |
|  |  | 52 | P16109 | 169 | hsa05142 |
|  |  | 53 | Q07869 | 170 | hsa05216 |
| **No.** | **Compound** ^a^ | **No.** | **Target** ^b^ | **No.** | **Pathway** ^c^ |
|  |  | 54 | Q03181 | 171 | hsa04931 |
|  |  | 55 | P37231 | 172 | hsa05161 |
|  |  | 56 | P16581 | 173 | hsa04261 |
|  |  | 57 | P14679 | 174 | hsa05221 |
|  |  | 58 | P06239 | 175 | hsa04150 |
|  |  | 59 | P11473 | 176 | hsa04914 |
|  |  | 60 | P47989 | 177 | hsa00910 |
|  |  | 61 | P15428 | 178 | hsa04370 |
|  |  | 62 | P56937 | 179 | hsa00590 |
|  |  | 63 | P05091 | 180 | hsa00232 |
|  |  | 64 | P00352 | 181 | hsa04921 |
|  |  | 65 | P35869 | 182 | hsa04024 |
|  |  | 66 | Q99700 | 183 | hsa04960 |
|  |  | 67 | Q9UNQ0 | 184 | hsa05205 |
|  |  | 68 | P53779 | 185 | hsa00380 |
|  |  | 69 | P0DP23 | 186 | hsa05031 |
|  |  | 70 | Q92887 | 187 | hsa04750 |
|  |  | 71 | P00915 | 188 | hsa00860 |
|  |  | 72 | O43570 | 189 | hsa05140 |
|  |  | 73 | Q16790 | 190 | hsa05218 |
|  |  | 74 | P16152 | 191 | hsa05220 |
|  |  | 75 | O75828 | 192 | hsa04910 |
|  |  | 76 | O95067 | 193 | hsa05133 |
|  |  | 77 | P05177 | 194 | hsa04930 |
|  |  | 78 | Q16678 | 195 | hsa05030 |
|  |  | 79 | P11509 | 196 | hsa05145 |
|  |  | 80 | P20813 | 197 | hsa04621 |
|  |  | 81 | P33261 | 198 | hsa00591 |
|  |  | 82 | P11712 | 199 | hsa01100 |
|  |  | 83 | Q99714 | 200 | hsa05206 |
|  |  | 84 | P14061 | 201 | hsa05203 |
|  |  | 85 | P37059 | 202 | hsa04510 |
|  |  | 86 | Q04760 | 203 | hsa05202 |
|  |  | 87 | P63092 | 204 | hsa04972 |
|  |  | 88 | Q03164 | 205 | hsa04713 |
|  |  | 89 | P14151 | 206 | hsa04066 |
|  |  | 90 | B2RXH2 | 207 | hsa05034 |
|  |  | 91 | P10636 | 208 | hsa05152 |
|  |  | 92 | P28482 | 209 | hsa05211 |
|  |  | 93 | Q16236 | 210 | hsa04310 |
|  |  | 94 | P08183 | 211 | hsa03320 |
|  |  | 95 | P19793 | 212 | hsa04550 |
|  |  | 96 | P11309 | 213 | hsa04662 |
| **No.** | **Compound** ^a^ | **No.** | **Target** ^b^ | **No.** | **Pathway** ^c^ |
|  |  | 97 | Q9Y3R4 | 214 | hsa04920 |
|  |  | 98 | Q13285 | 215 | hsa04620 |
|  |  | 99 | Q16637 | 216 | hsa04520 |
|  |  | 100 | P22309 |  |  |
|  |  | 101 | Q9HAW8 |  |  |
|  |  | 102 | Q9HAW9 |  |  |
|  |  | 103 | P22310 |  |  |
|  |  | 104 | P54855 |  |  |
|  |  | 105 | O95271 |  |  |
|  |  | 106 | P27986 |  |  |
|  |  | 107 | Q9NPH5 |  |  |
|  |  | 108 | P27361 |  |  |
|  |  | 109 | Q9H2K2 |  |  |
|  |  | 110 | O75164 |  |  |
|  |  | 111 | Q9H3R0 |  |  |
|  |  | 112 | P09923 |  |  |
|  |  | 113 | Q9P0U3 |  |  |
|  |  | 114 | P55072 |  |  |
|  |  | 115 | P49674 |  |  |
|  |  | 116 | P18031 |  |  |
|  |  | 117 | P49840 |  |  |
|  |  | 118 | O60674 |  |  |
|  |  | 119 | O15530 |  |  |
|  |  | 120 | P07900 |  |  |
|  |  | 121 | Q13547 |  |  |
|  |  | 122 | O15379 |  |  |
|  |  | 123 | Q13526 |  |  |
|  |  | 124 | Q15831 |  |  |
|  |  | 125 | P09874 |  |  |
|  |  | 126 | Q05655 |  |  |
|  |  | 127 | P00533 |  |  |
|  |  | 128 | P17252 |  |  |
|  |  | 129 | P07237 |  |  |
|  |  | 130 | P14410 |  |  |

a, Potential *α*-glucosidase inhibitors screened from flavonoids of *Radix Scutellariae*; b, Names of the target proteins are uniformed by Uniprot; c, Names of the pathways are uniformed by KEGG.
